# Complex anionic and zwitterionic N-glycans in *Drosophila melanogaster*☆

**DOI:** 10.1016/j.bbagen.2026.130961

**Published:** 2026-05-08

**Authors:** Kristína Mad’arová, Zuzanna Dutkiewicz, Lena Nuschy, Maria Magdalena John, Huijie Wang, Barbara Eckmair, Iain B.H. Wilson, Katharina Paschinger

**Affiliations:** Institut für Biochemie, Universität für Bodenkultur, 1190 Wien, Austria

**Keywords:** Drosophila, Glycomics, Phosphoethanolamine, Mass spectrometry

## Abstract

*Drosophila melanogaster* is arguably the most widely-employed invertebrate model organism; here we have focussed on its anionic N-glycome applying our off-line HPLC-MALDI-TOF-MS approach. Using MS/MS in combination with chemical and enzymatic treatments, we reveal not only sulphated and glucuronylated N-glycans further to those previously proposed, but demonstrate that this dipteran insect modifies some structures with phosphoethanolamine, a zwitterion. Although not as complex as some other insect N-glycomes, N-glycans with up to three antennae can feature also LacdiNAc or β1,3-galactosylation as the basis for anionic modifications, whereby some glycans also carry up to five anionic/zwitterionic moieties; in total approximately 180 N-linked oligosaccharide structures, including isomers and isobars, can be proposed. A prototype microarray based on fractionated N-glycans was printed and showed different fucose-specific lectin binding to N-glycans from a wild-type and a fucose-deficient strain, whereas serum amyloid P bound anionic glycans. In terms of enzymatic analyses, recombinant forms of both *N*-acetylglucosaminyltransferases II and IV were expressed and their activities correlate with the occurrence of up to three antennae on *Drosophila* N-glycans. Overall, in keeping with the accepted phylogeny, the fruit fly is indeed closest in glycomic terms to mosquitoes, but also shares the feature of phosphoethanolamine modifications with the honeybee, rather than the phosphorylcholine found in lepidopteran species. Nevertheless, there are many unanswered questions regarding N-glycosylation in *Drosophila*; a mix of genomic, biochemical and glycomic approaches will be required to solve the biosynthesis and functions of this key set of post-translational modifications in this and other insects.

## 1 Introduction

*Drosophila melanogaster* is one of the most familiar model organisms based on a long tradition of genetic research; thus, it is no surprise that it has also been employed in glycobiological research for a number of decades [1,2]. Even amongst the fly mutants studied by Morgan one hundred years ago, there are some with a glyco-relevance, including rotated abdomen (rt) and Notch (N) [3,4], which were later revealed to respectively correspond to a glycosyltransferase and a glycoprotein [5,6].

The N-glycans of *D. melanogaster* were first studied in the 1990s [7], whereby the belief gained hold that their structures were a relatively simple series of pauci- and mannosidic structures. The basics of the N-glycosylation pathway, including all the steps in the ER and those as far as transfer of *N*-acetylglucosamine residues in the Golgi, are conserved as compared to other animals, including mammals [8]. In the 2000s, core difucosylated N-glycans as well as glucuronylated structures were first found [9–11]. Sialylation of fly glycans has also been proposed [12], whereby the early report of polysialic acid [13] is now considered to be incorrect. Similarly to the N-glycans, only simple O-glycans were first reported in *D. melanogaster* [14], but more recently sulphation, glucuronylation and modification with phosphoethanolamine have also been revealed on O-GalNAc and/or O-fucose [15–17]. N-glycans and the various forms of O-glycans have a number of significant impacts on the development and physiology of the fly, including in the neural and innate immune systems [1,18]. Glycosaminoglycans (chondroitin and heparan sulphates) are also developmentally very important in the fruit fly [19,20], while the structures, early biosynthesis and some functions of fly glycosphingolipids have also been elucidated [21].

In the past decade and by employing optimised workflows, we have shown that invertebrate N-glycomes are indeed rather complex, also in insects [22]. Variations in the occurrence of zwitterionic modifications (phosphoethanolamine and phosphorylcholine) between species have been revealed, whereas all insects studied to date possess sulphated and glucuronylated N-glycans [23]. In this context, we embarked on a reappraisal of the N-glycome of *D. melanogaster*, focussing on comparing the head, body and whole adult fly N-glycomes of a wild-type strain. We also developed a prototype fly glycan array and proved the activity of two key glycosyltransferases required for synthesis of tri-antennary N-glycans. Thereby, we reveal that *D. melanogaster* has an extensive repertoire of anionic N-glycans with not only sulphated and glucuronylated glycans, but also ones modified with phosphoethanolamine, also in the context of difucosylated cores. Thus, the fruit fly N-glycome is more complicated than previously thought, raising the question as to which structures are specifically responsible for the neural phenotypes which occur upon ablation of complex N-glycan biosynthesis.

## 2 Methods

### 2.1 Glycan preparation

*Drosophila melanogaster* strain Tf24 was maintained in the laboratory; heads were separated from bodies using a scalpel and frozen. The sub-sequent N-glycan extraction and purification was based on previous procedures [24]; a workflow is shown in Supplementary Fig. 1. Fruit fly material (either 3 g body, 0.6 g head or 1 g whole fly, wet weight) was ground and dissolved in 15 mL of 50 mM ammonium bicarbonate at pH 8, and the mixture was homogenized by boiling while stirring. CaCl_2_ was added to achieve a final concentration of 0.5 mM, followed by the addition of 3 mg of thermolysin, and the mixture was incubated for 3 h at 70 °C. The supernatant was then acidified to below pH 4 and incubated with Dowex cation exchange resin (Biorad, AG 50 W × 8; 10 mL) for 30 min before being loaded into a 20 mL column. The column was washed with six column volumes of 2% (*v*/v) acetic acid and glycopeptides eluted with three column volumes of 0.5 M ammonium acetate, pH 6, into fractions of 1.8 mL each. The orcinol-positive glycan-containing fractions were lyophilized.

The sample was re-dissolved in 0.5 mL of 0.5% (*v*/v) acetic acid and applied to a G25 gel filtration column (80 ml). Orcinol-positive fractions were pooled, lyophilized, and reconstituted in 100 mM ammonium bicarbonate, pH 8, then treated with 3 μL of PNGase F (Roche, 14740121) at 37 °C for 6 h. After heat inactivation, the sample was adjusted to pH 4 using 1 M ammonium acetate, and recombinant PNGase A (NEB, P0707S) was added (37 °C overnight). The digest was adjusted to 2% (*v*/v) acetic acid and loaded onto a second Dowex cation exchange column (5 mL) as previously described. Free glycans were eluted with 2% (v/v) acetic acid, pooled and lyophilized. Alternatively, the PNGase F digested material was immediately loaded onto the second Dowex column as above and the PNGase F-released glycans eluted first; the remaining glycopeptides were eluted with 0.5 M ammonium acetate, pH 6, desalted by gel filtration and treated with PNGase A, prior to loading onto a third Dowex column (5 mL) and elution of the PNGase A-released glycans (see workflow in Supplementary Fig. 2).

The glycans were then dissolved in 50 μL of water and loaded onto a home-packed non-porous graphite carbon column (ENVICarb). The column was washed with water and glycans eluted with 40% (*v*/v) acetonitrile (the neutral fraction), followed by 40% (v/v) acetonitrile with 0.1% (v/v) trifluoroacetic acid (the anionic fraction). The eluents were lyophilized and further purified using a home-packed C18 column. After application of the glycans, the column was washed with water and subsequently eluted in a stepwise manner with 15%, 40%, and 100% (*v*/v) methanol. The fractions were dried under vacuum and analyzed by MALDI-TOF mass spectrometry to determine glycan-containing fractions prior to 2-aminopyridine (PA) labeling.

### 2.2 Glycan analysis

The procedure for PA labelling has been previously published [24]. In brief, 2-aminopyridine (Pestanal, Sigma, 99%) was dissolved in a 1:2 mixture of concentrated HCl/water to yield a slightly basic solution and mixed with the lyophilized sample. This solution was boiled for 10 min, cooled, and then NaCNBH_3_ dissolved in PA/HCl/water was added. After incubation overnight at 90 °C, excess labelling reagent was removed using a G15 gel filtration column. The fractions containing PA-labelled glycans were combined and lyophilized overnight. The neutral and anionic PA-labelled glycans were separated by HPLC using a RP amide column (Supelco, Ascentis Express RP-Amide, 150 cm × 4.6 mm; 0.8 mL/min). Buffer A consisted of 0.1 M ammonium acetate, pH 4, while Buffer B was 30% (v/v) methanol, with the following gradient: 0–4 min at 0% B; 14 min at 5% B; 24 min at 15% B; 34 min at 35% B; and 35–40 min back to 0% B [24,25]. Fractions were collected manually based on fluorescence intensity (excitation/emission 320/400 nm), lyophilized overnight, and then reconstituted in 5–10 μL of water. For the head PNGase F-released anionic glycome, glycans were fractionated on a Kinetex RP-column (Phenomenex® XB-C18 100 A, 5 μ, 250 × 4.6 mm) using the following gradient [26]: buffer C (0.1 M ammonium formate, pH 6) and buffer B with a flow rate of 0.8 mL/min: 0–5 min, 0–10% B; 5–35 min, 10–40% B; 35–40 min, 40–50% B; 40–45 min, 50–65% B; 45–49 min, hold at 65%; 49–51 min, return to starting conditions; the fractions were stored at −20 °C. The retention time trends for different glycan structural types on RP-HPLC were originally described by Tomiya et al., e.g., effects of oligomannosidic isomers, core fucosylation, antennae or GalNAc modification [27–29], and have been observed by us using fused core columns in other studies [30,31].

For MALDI-TOF MS analysis (Rapiflex, Bruker), 0.5 μL of the sample was spotted and dried under vacuum before applying 0.5 μL of 3 mg/ml 6-aza-2-thiothymine (ATT in 10 mM ammonium citrate in 50% ethanol; Alfa-Aesar, Thermo Scientific) and drying again. Peaks with potential glycan masses were fragmented in LIFT mode without collision gas and data were assessed manually. For anionic fractions, both positive and negative modes in reflector mode are used for MS scanning (*m/z* 700–3500) and MS/MS fragmentation. For details refer to “Further information regarding the glycomic analyses” in the Supplementary Information.

Exoglycosidases utilized to verify glycan structures included: jack bean β-*N*-acetylhexosaminidase (Sigma, A2264), bovine α-fucosidase (Sigma, F5884), human β-glucuronidase (Biotechne, 6144-GH-020), jack bean α-mannosidase (NEB, P0768), and *Xanthomonas manihotis* β1,3-galactosidase (NEB, P0726S). Digestion was carried out in 20 mM ammonium acetate, pH 4–5. The digests were then directly spotted for MALDI-TOF analysis. For hydrofluoric acid treatment, 4 μL of HF (Sigma, 48%, ACS reagent) was added to each dried glycan fraction and incubated on ice in a cold room for 48 h. The digest was then dried using a SpeedVac, re-dissolved in water, and subsequently spotted for mass spectrometry analysis.

### 2.3 Glycan array

Neutral and anionic glycans of *Drosophila* body samples of Canton S and *nac1* strains were enriched using solid-phase extraction on non-porous graphitized carbon (SupelClean ENVICarb, Sigma-Aldrich) [32] and labelled with 2-amino-*N*-(2-amino-ethyl)-benzamide (AEAB) as described [30,33]. In brief, dried glycans were dissolved in 0.35 M AEAB and 1 M NaCNBH_3_ and incubated for 2 h at 65 °C. After washing of excess AEAB label, glycans were purified by normal-phase HPLC (Zorbax NH2 Column 4.6 × 250 mm, 5-μm with guard column 4.6 × 12.5 mm 5-μm; Agilent Technologies, Inc.; 0.8 mL/min flow rate, 80% acetonitrile in 125 mM ammonium acetate buffer pH 4.5; with fluorescence detection using a Shimadzu RF-20 A XS Fluorescence Detector; excitation/emission of 330/420 nm) and afterwards separated by reversed-phase HPLC (Kinetex LC column 5 μm XB-C18 100 Å, 250 × 4.6 mm with SecurityGuard ULTRA Holder; Phenomenex Inc.; 0.8 mL/min flow rate; solvent A: 0.1 M ammonium acetate buffer pH 4; solvent B: 30% MeOH. Conditions for neutral glycans: 0–30 min, 0–30% B; 30–35 min, 40% B; 35–40 min, 58% B; 40–44 min, 58% B; 44–46 min, 0% B; 46–51 min, 0% B. Conditions for anionic glycans: 0–2 min, 0–15% B; 2–5 min, 25% B; 5–10 min, 30% B; 10–20 min, 50% B; 20–35 min, 95% B; 35–40 min, 95% B; 40–46 min, 0% B; 46–51 min, 0% B.) HPLC-Fractions were analyzed by MALDI-TOF MS and MS/MS on a Bruker Autoflex Speed instrument, pooled and normalized according to their peaks’ fluorescence intensities. Neutral glycan pools of the two strains were kept separate while, due to the relatively low amounts, anionic fractions were combined. The pools were printed in replicates of five onto NHS-derivatised Nexterion H glass slides (Schott, Jena, Germany), the slides processed, and assays performed as in previous studies [30,33,34]. The biotinylated plant lectins *Aleuria aurantia* lectin (AAL, 2 mg/ml), concanavalin A (ConA, 5 mg/ml), *Sambucus nigra* lectin (SNA, 2 mg/ml), *Maackia* lectins (MAL I and MAL II; 2 mg/ml) and wheat germ agglutinin (WGA, 5 mg/ml), all Vector Laboratories, were applied in 1:100 dilution and detected with AlexaFluor 647 streptavidin (2 μg/ml; Invitrogen, ThermoFisher). Incubation with serum amyloid protein (1:200 diluted; Fitzgerald) was followed by anti-SAP from rabbit (1:1000 diluted; Calbiochem) and finally AlexaFluor647 anti-rabbit IgG (2 μg/ml; Invitrogen, ThermoFisher). Fc-tagged *Aedes* lectins aeCTL23, aeCTLGA6a, aeCTLMA14 and aeMosGCTL-1 ^(*35*)^ were the kind gift from Dr. Bernd Lepenies (LMU München), applied 1:100 diluted and detected with murine anti-human IgG Fc (1:1000 diluted, BioLegend) and Alex-aFluor 647 anti-mouse antibody (2 μg/ml; Invitrogen, ThermoFisher). Slides were scanned with GenePix 4400 A (Molecular Devices, LLC) at 635 nm with maximum gain. Data were analyzed with the respective GenePix Version 7 software, the total fluorescence intensity values were background subtracted and evaluated in Microsoft Excel and GraphPad Prism. For details on buffers, concentrations and settings refer to “Further information regarding the glycan array analyses” in the Supplementary Information.

### 2.4 Expression and assay of recombinant glycosyltransferases

For cloning into an insect expression vector, the full open reading frame for the p61 alternative transcript of *D. melanogaster N*-acetylglucosaminyltransferase II [36] was isolated by PCR using Expand High Fidelity polymerase (Roche), p61 plasmid DNA (the kind gift of Dr. Tsitilou, University of Athens, Greece) and the following primers: DmMGAT2/fwd, CGGGGTACCATGGGTCGCAAGCGCAACA; DmMGAT 2/rev, GCTCTAGATTACCTCGTGGCCAGCGT. Both pPIZT/V5-His and the PCR product were cut with *Eco*RI and *Kpn*I prior to ligation and subsequent transformation of *E. coli*. Clones were screened by PCR with OpIE/fwd and OpIE/rev primers and positive clones were verified by sequencing with the BigDye® Terminator v3.1 Cycle Sequencing Kit and analysis with an ABI sequencing instrument. *T. ni* HighFive cells were transiently transfected by using Cellfectin® (Invitrogen) and transfection efficiency shown by fluorescence microscopy (the plasmid features a reading frame encoding a GFP fusion protein). Although the full length form was transfected, enzyme activity was detected in the culture supernatant, but not in the case of an empty vector control.

The open reading frame for the *D. melanogaster N*-acetylglucosaminyltransferase IVa (CG9384) excluding the 5′-end encoding the cytosolic and transmembrane domains was synthesised. The synthetic gene sequence was designed with overhangs for direct cloning via HiFi DNA Assembly Cloning Kit (NEB) after the N-terminal 8xHis-tag and TEV cleavage site in the pTT22SSP4 vector (Canadian National Research Council). HEK293-6E cells were cultivated in FreeStyle F17 Expression Medium (Gibco) supplemented with 4 mM L-glutamine (Gibco), 0.1% Pluronic F-68 (Gibco) and 25 μg/ml Geneticin selective antibiotic (Gibco). The cells were maintained on an orbital shaker at 125 rpm in hydrated atmosphere with 5% CO2 at 37 °C and passaged at 2–3 days intervals; cells were transfected using polyethyleneimine and cultivated for 4–5 days till harvest. They were kept on 37 °C directly after transfection, with a shift to 32 °C 24 h after transfection and the addition of 0.5% tryptone N1 48 h after transfection. The supernatant was harvested by centrifugation at 2500 *g* for 15 min at 4 °C and filtered (0.45 μm) prior to application to a 1 ml HisTrap Excel column (Cytiva). The equilibration buffer comprised 25 mM sodium phosphate and 500 mM NaCl at pH 7.4. The wash buffer contained additionally 20 mM imidazole, and the elution buffer contained 500 mM imidazole. Relevant fractions were pooled, concentrated and rebuffered into 25 mM Tris-Cl, 150 mM NaCl, pH 7.5 (MWCO 10 kDa; Sartorius). Expression was shown by SDS-PAGE and Western blotting.

*N*-acetylglucosaminyltransferases II and IVa were assayed using respectively MGn or GnGn dabsylated glycopeptides derived from asialoagalactofibrin as substrates [9]. In the standard assays, approximately 0.1 nmol glycopeptide was incubated with 0.5 μl enzyme solution (0.5 mg/ml), 10 mM UDP-GlcNAc (Sigma-Aldrich; alternatively UDP-[^13^C]-GlcNAc from Omicron Biochemicals), 20 mM MnCl_2_ (or other cation or EDTA) and 2-amino-2-methyl-1,3-propanediol (AMPD) buffer, pH 7.0, for 30 mins, 30 °C, prior to MALDI-TOF MS analysis with α-cyanohydroxycinnamic acid as matrix on either a Bruker Ultraflex (MGAT2 assays in 2009) or Bruker Rapiflex (MGAT4 assays in 2025).

## 3 Results

### 3.1 Neutral N-glycans

**Fig. 1:**
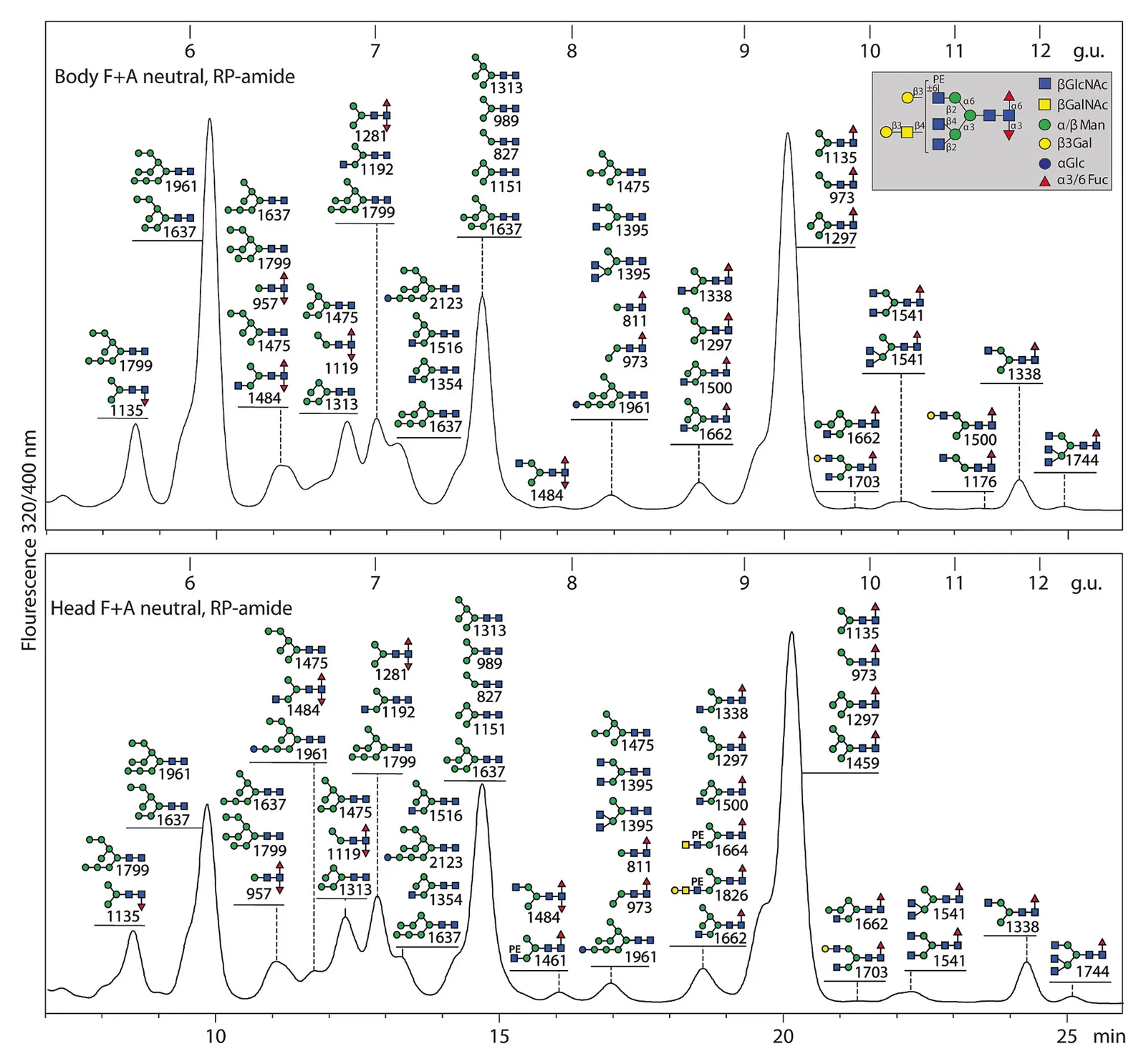
RP-HPLC of Drosophila neutral N-glycans. RP-amide chromatograms of neutral N-glycans released by PNGase F followed by PNGase A for Tf24 bodies and heads, eluted in the acetonitrile fraction from non-porous graphitised carbon prior to labelling with 2-aminopyridine, were calibrated in terms of glucose units (g.u.). Fractions are annotated with proposed structures using the Symbolic Nomenclature for Glycans (see inset) with the most dominant structure uppermost on the basis of MALDI-TOF MS/MS and retention times as compared to other insect glycans. For MALDI-TOF MS of the entire pools, a workflow and a comparison to the HPLC profile for whole flies, refer to Supplementary Figs. 1 and 3. Example MS/MS are available via Glycopost.

We prepared glycopeptides from *Drosophila melanogaster* Tf24 whole adults as well as from the heads separated from the bodies; the preparations were treated with PNGase F followed by PNGase A, subject to solid phase extraction on non-porous graphitised carbon and fractionated into neutral and anionic pools prior to fluorescent labelling with 2-aminopyridine and RP-amide HPLC. The profiles for the three samples were rather similar with around 50 defined structures, although with small variations (Fig. 1 and Supplementary Fig. 3). As in previous studies [9], the main detected structures are the paucimannosidic, oligomannosidic and hybrid/pseudohybrid structures typical of insects, including some core difucosylated glycans eluting at 6.5–7 g.u., which were also found in Canton S adults and which were dominant in the subpool of glycans released by PNGase A alone (Supplementary Figs. 2 and 4). In addition, three glycans in neutral pools were detected in both positive and negative modes with a mass increment of 123 Da, suggestive for a modification with phosphoethanolamine (7.8–8.8 g.u.; see below) as also found on honeybee N-glycans [30]; one neutral glycan with the same late retention time (13 g.u.) and similar fragmentation pattern as a triantennary structure in mosquito samples [16] was also present (Fig. 1), reflecting the glycogenomic capacity to synthesise triantennary glycans.

### 3.2 Anionic N-glycans

**Fig. 2:**
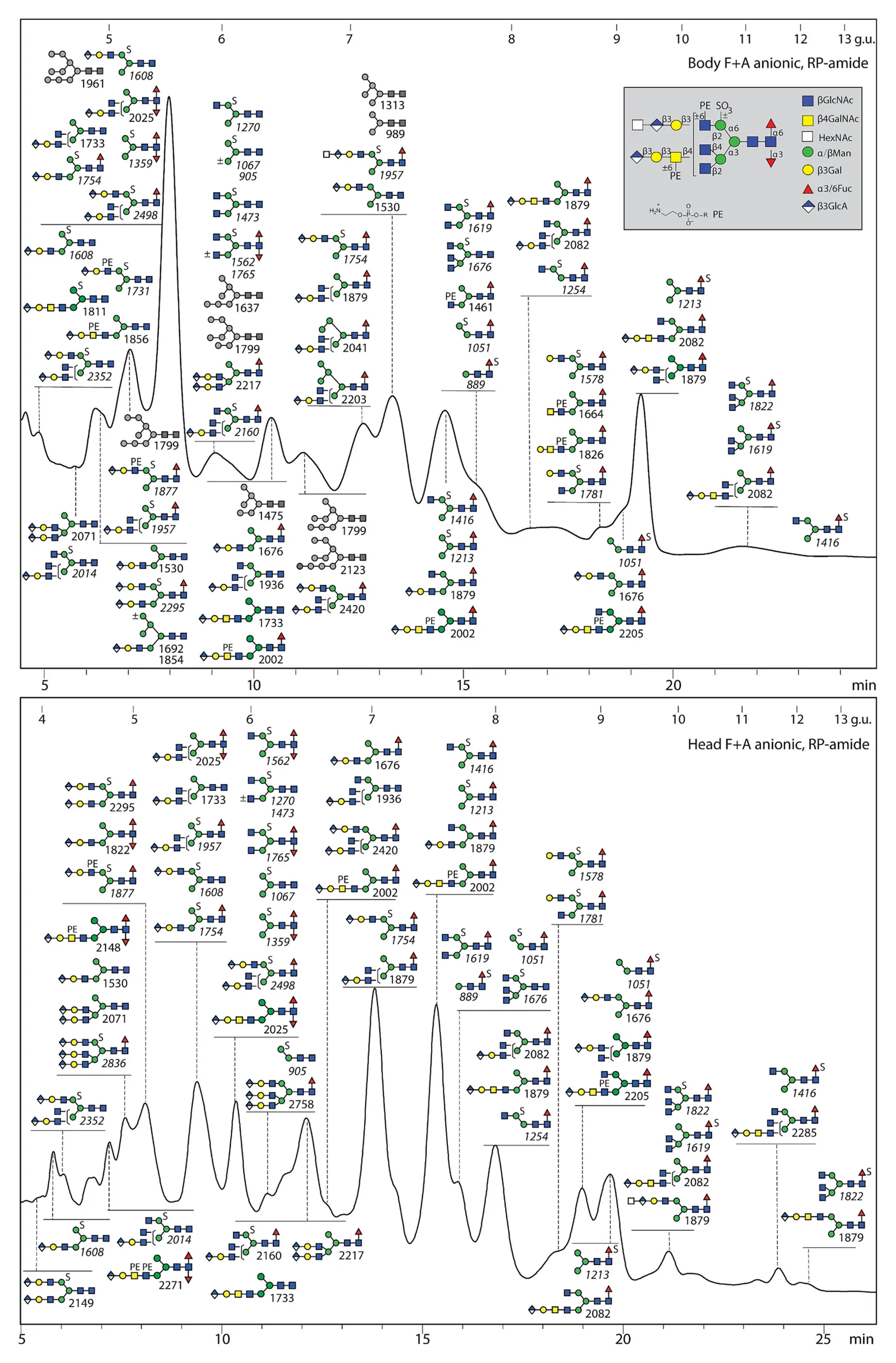
RP-HPLC of Drosophila anionic N-glycans. RP-amide chromatograms of anionic N-glycans released by PNGase F followed by PNGase A for Tf24 bodies and heads, eluted in the 0.1% trifluoroacetic acid fraction from non-porous graphitised carbon prior to labelling with 2-aminopyridine, were calibrated in terms of glucose units (g.u.). Fractions are annotated with proposed structures using the Symbolic Nomenclature for Glycans (see inset) with the most dominant structure uppermost on the basis of MALDI-TOF MS/MS (for examples, see Figs. 3–5 as well as mzxml files available via Glycopost) and comparative retention times; *m*/*z* values in italics are for negative mode of sulphated structures. While three anionic Man_4–5_-type hybrid structures were detected only in bodies, more difucosylated ones were defined in the head sample. For MALDI-TOF MS of the entire pools, a workflow and a comparison to the HPLC profile for whole flies, refer to Supplementary Figs. 2 and 3. Co-eluting oligomannosidic structures are shown in grey.

**Fig. 3:**
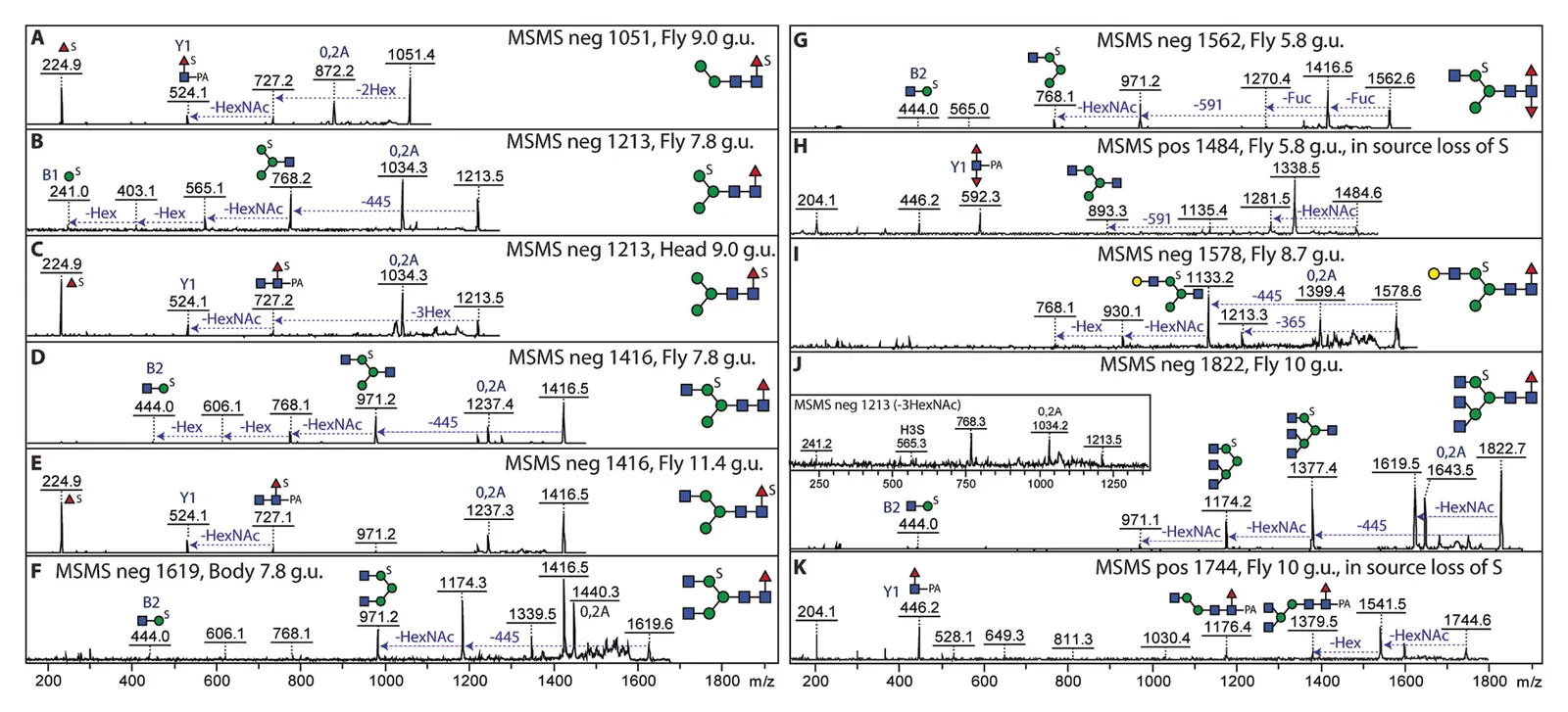
Mass spectrometric analysis of Drosophila sulphated N-glycans. RP-HPLC fractionated anionic N-glycans (see Fig. 2 and Supplementary Fig. 3) were subject to negative mode MALDI-TOF MS/MS; key fragments at *m/z* 225 and 524 show sulphation of core α1,6-fucose (A, C, E), while B ions at, e.g., *m/z* 241, 444, 606, 768 and 971 correlate with sulphation of the α1,6-mannose residue (B, D, F, G, I and J). Negative mode MS/MS also generally results in a ^0,2^ A cross-ring cleavage of the reducing terminal GlcNAc or a loss of the mono-or difucosylated reducing terminal residue (losses of 445 or 591). Jack bean β-hexosaminidase digestion of the triantennary *m/z* 1822 structure results in loss of three HexNAc residues and a shift in the MS/MS spectrum (compare inset in J with spectrum in B); positive mode MS/MS of the [M-SO_3_]^+^ ions, resulting from in source loss of sulphate, shows the underlying core fucosylation status with Y1 ions at *m/z* 446 or 592 (*H*,*K*). The largest structure in each panel represents the overall glycan, while the smaller ones represent key fragment ions.

In terms of fluorescence intensity, the anionic pools correspond to about 5% of the total N-glycomes and the RP-amide chromatograms for all three anionic pools feature around 60 structures as judged by MS/MS in combination with selected chemical or glycosidase treatments and comparison to retention times in previous studies on insect N-glycans (Fig. 2 and Supplementary Fig. 3). As with other insects and as indicated by previous permethylation data on a *Drosophila* pseudohybrid structure [16], glycans with sulphation of fucose or mannose were detected; the characteristic negative mode MS/MS fragments at *m/z* 225, 524 (FucS and FucSGlcNAc-PA for sulphation of fucose), 241 or 444 (HexS or HexSHexNAc for sulphation of mannose) aided definition of isomeric structures (Fig. 3). LC-MS/MS data on a lepidopteran protease are compatible with either a 4- or 6-sulphation of mannose [37], but the exact linkage for sulphation of fucose is unknown. No sulphation of the outer antennae was observed as judged by the lack of corresponding fragments (e.g., HexNAcHexAS, as found in mosquito larvae [16]).

**Fig. 4:**
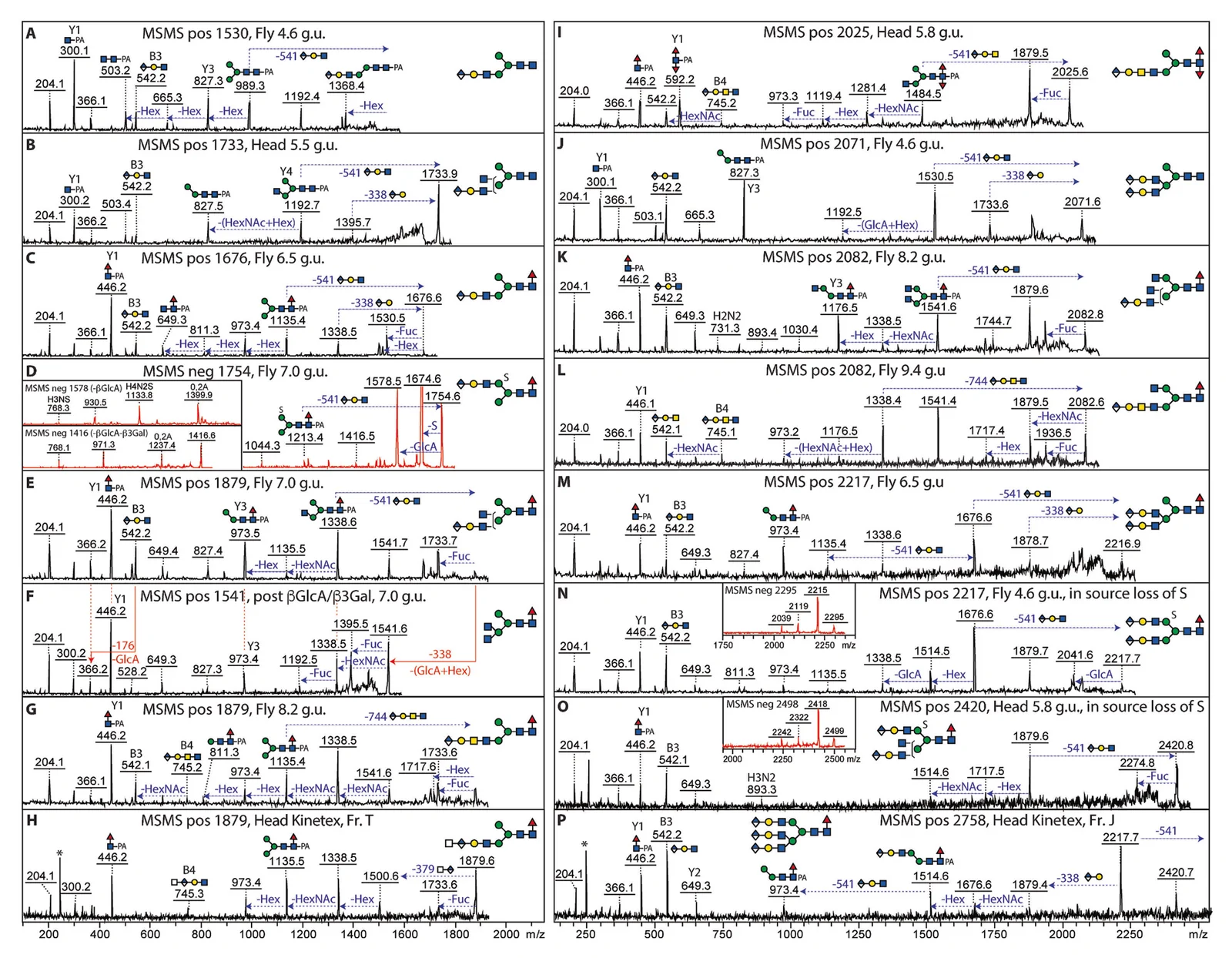
Mass spectrometric analysis of Drosophila glucuronylated N-glycans. RP-HPLC fractionated anionic N-glycans (see Fig. 2 and Supplementary Fig. 3) were subject to positive and negative mode MALDI-TOF MS/MS. Glycans with only glucuronic acid as the anionic moiety were best fragmented in positive mode, whereby the key B3 or B4 ions at *m/z* 542 and 745 are indicative for the terminal modification with either GlcAβ1,3Galβ1,3GlcNAc (A-C, E, J, K, M-P), GlcAβ1,3Galβ1,3GalNAcβ1,4GlcNAc (G, I, L) or an undefined HexNAc capping of GlcAβ1,3Galβ1,3GlcNAc (H), while Y1 ions at *m/z* 300, 446 and 592 show the fucosylation status of the reducing terminal GlcNAc residue (Fuc_0–2_GlcNAc_1_-PA). Glycans carrying both sulphate and glucuronic acid were fragmented in negative mode (panel D, and insets in N and O, in red), whereby primarily neutral losses of sulphate or glucuronic acid were observed. Combined digestion with human β-glucuronidase (βGlcA) and *Xanthomonas* β1,3-specific galactosidase (β3Gal) resulted in a loss of 338 Da (insets in D and panel F), ruling out that the sulphate on the *m/z* 1754 glycan (panel D) is on the antennal glucuronic acid or galactose residues; also no outer antennal sulphated fragments were observed unlike mosquitoes. The largest structure in each panel represents the overall glycan, while the smaller ones represent key fragment ions. (For interpretation of the references to colour in this figure legend, the reader is referred to the web version of this article.)

Previously, ten glucuronylated mono- or bi-antennary N-glycan structures were shown in *Drosophila* OreR embryos [11]; here, we observed also tri-antennary and sulphated glycans with glucuronylated antennae as judged by the occurrence of the key B3 positive mode fragment at *m/z* 542 (Hex_1_HexNAc_1_HexA_1_; Fig. 4A-E and J, K, M-P). Example structures were digested by β-glucuronidase in combination with β1,3-specific galactosidase indicative of GlcAβ1,3Galβ1,3GlcNAc antennae (Fig. 4D and F). The definition of the glucuronic acid as being β1,3-linked is based on differential sensitivity of structures in other insects and nematodes to *E. coli* and *Helix pomatia* glucuronidases [30,34] as well as the previous MS/MS data showing a 1,3-linkage to the underlying hexose [11]. Some glycans displayed a B4 fragment at *m/z* 745, suggestive of a longer antenna (Fig. 4G, I and L), probably GlcAβ1,3Galβ1,3GalNAcβ1,4GlcNAc; alternatively, GlcAβ1,3Galβ1,3GlcNAc motifs capped with an unknown HexNAc were also detected as defined by B4 fragments at *m/z* 745 and neutral losses of HexNAc_1_HexA_1_ (i.e., 379 Da; see example in Fig. 4H). Both of these longer antennae are known from other insect species [16]. While glucuronylated glycans are detectable in positive and negative modes with MS/MS optimal in positive mode, the molecular ions for structures carrying both sulphate and glucuronic acid are observed rather only in negative mode; in MS/MS, the tendency is that loss of a sulphate is dominant and that there are few B ions (Fig. 4D, N and O and insets).

**Fig. 5:**
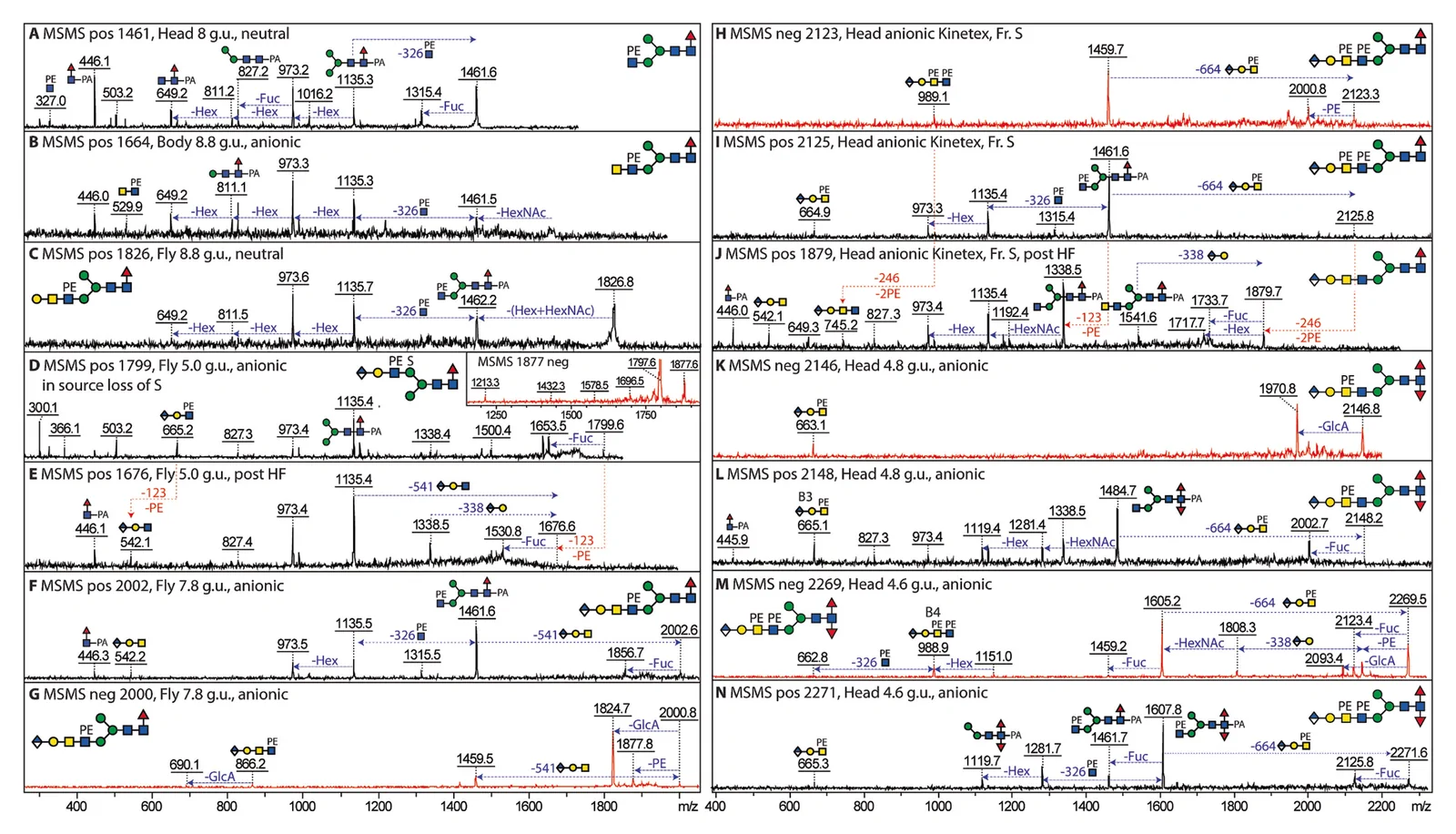
Mass spectrometric analysis of Drosophila phosphoethanolamine-modified N-glycans. RP-HPLC fractionated anionic N-glycans (see Fig. 2 for A-B and K–N, Supplementary Fig. 3 for C-G, Fig. 6 for H-J) were subject to positive (black) and negative (red) mode MALDI-TOF MS/MS. The dominant ions in the spectra result from neutral loss of the non-reducing terminal PE-containing motif (e.g., GlcNAcPE, GlcAGalHexNAcPE, GlcA alone or GlcAGalHexNAc) or of sulphate (inset in D), whereas PE-containing B ions at *m/z* 327, 530 and 665 in positive mode and at *m/z* 663, 866 and 989 in negative mode are of lower intensity. Hydrofluoric acid treatment results in loss of PE (–123 Da; see panels E and J). For further MS/MS of difucosylated glycans modified by phosphoethanolamine, refer to Supplementary Fig. 2C; for further PE-modified structures separated on the Kinetex column, refer to Supplementary Fig. 5. (For interpretation of the references to colour in this figure legend, the reader is referred to the web version of this article.)

The final category of ‘charged’ N-glycans are those with a phosphodiester-based modification of 123 Da, i.e., phosphoethanolamine. MS/MS spectra were dominated by neutral losses from the antennae (e.g., loss of 326 Da, i.e., HexNAc_1_PE), but the B ions were of generally lower intensity, regardless of the MS mode. While as mentioned above some of these were detected in both the neutral and anionic pools (Fig. 5 A-C), those with an additional glucuronic acid and even sulphate were found only in the anionic pools (Fig. 5D, F and G). Hydrofluoric acid treatment resulted in loss of the 123 Da modification (Fig. 5E), resulting in conversion of B ions at *m/z* 665 (Hex_1_HexNAc_1_HexA_1_PE) to ones at *m/z* 542 (i.e., Hex_1_HexNAc_1_HexA_1_). Structures with longer antennae were also observed which could carry one or two phosphoethanolamine residues as well as core difucosylation. These structures were detected in both positive and negative modes, whereby B ions at *m/z* 663 (Hex_1_HexNAc_1_HexA_1_PE) and 989 (Hex_1_HexNAc_2_HexA_1_PE_2_) in negative mode as well as the neutral loss of 664 Da defined the locations of the HF-sensitive phosphoethanolamine residues to the putative LacdiNAc unit (Fig. 5H-N). This zwitterionic modification was most easily detected in the head samples, as judged by the initial MALDI-TOF MS profile (Supplementary Fig. 1) and by the number of PE-modified glycans detected in the HPLC fractions (Fig. 2). When analysing the PNGase A released (post PNGase F) anionic N-glycans (Supplementary Fig. 2B and C), a further six zwitterionic structures (*m/z* 1607, 1810, 1972, 2095, 2148 and 2271) not found in the pool resulting after only PNGase F release were detected. Reanalysis of selected fractions of Canton S adults from twenty-five years ago [9] also showed the presence of sulphated, glucuronylated and phosphoethanolamine-modified glycans (Supplementary Fig. 4).

**Fig. 6:**
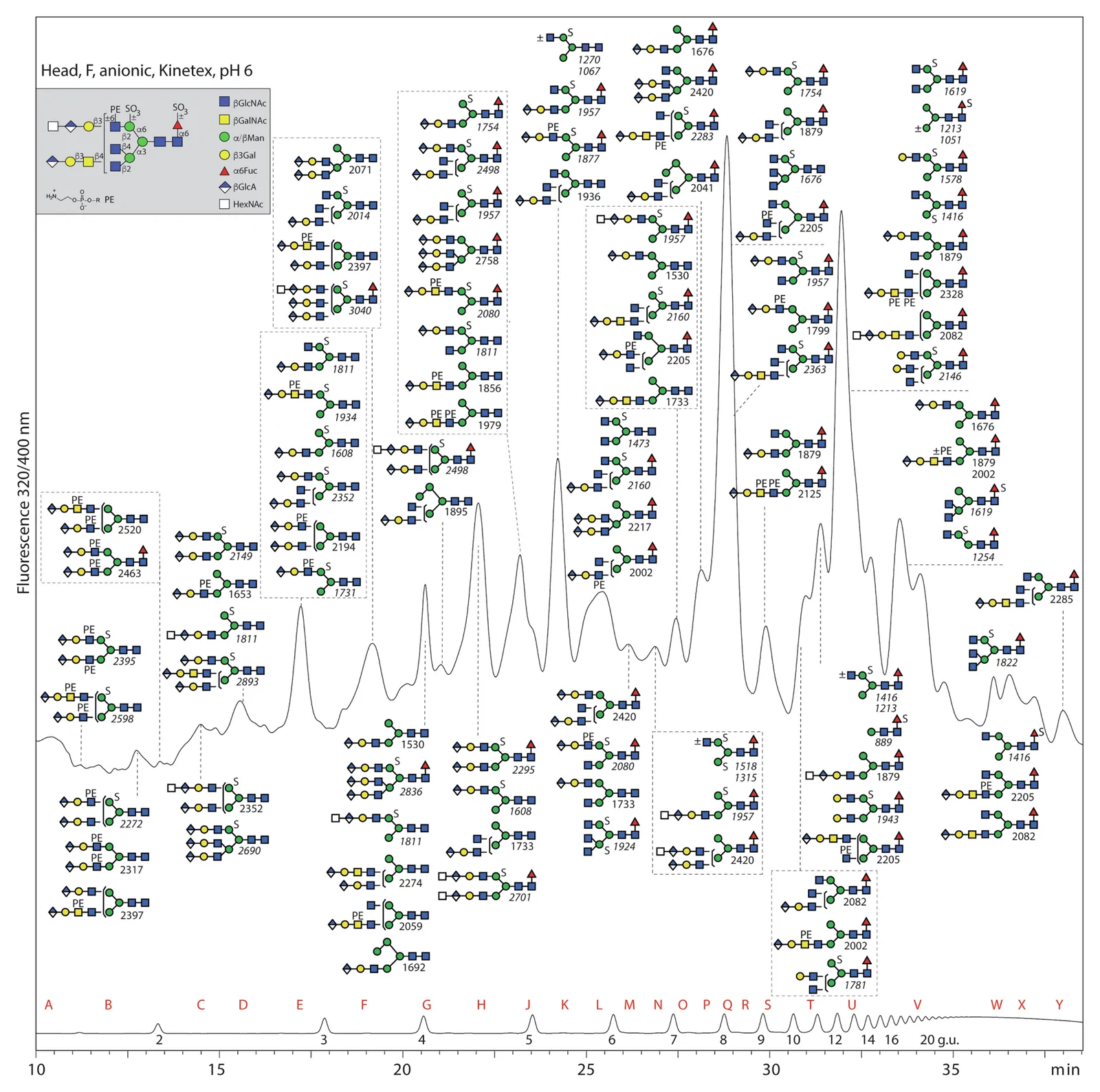
RP-HPLC analysis of anionic N-glycans from Drosophila heads. PNGase F-released anionic N-glycans were fractionated on a Kinetex column using a gradient of methanol in ammonium acetate, pH 6 buffer. For the workflow and the MS/MS of the annotated glycans, refer to Supplementary Figs. 2 and 5; refer also to 100 mzxml files available via Glycopost. Fractions are labelled A-Y and are annotated on the basis of MALDI-TOF MS/MS using the Symbolic Nomenclature for Glycans (see inset) with the most dominant proposed structure uppermost.

As sialylation has been proposed as a modification of fruit fly N-glycans on the basis of MS analyses and a characterised α2,6-sialyltransferase [12,38], we also employed a modified HPLC method previously used with 2-aminopyridine-labelled sialylated N-glycomes from echinoderms [26]. With a solvent system based on pH 6, rather than pH 4, acidic conditions during post-fractionation lyophilisation which can impair detection of intact sialylated glycans are thereby circumvented. We used this with PNGase F released anionic N-glycans from Drosophila Tf24 heads and each fraction was analyzed in both positive and negative MALDI-TOF MS modes. While we found a series of sulphated, glucuronylated and phosphoethanolamine-modified N-glycans, no masses for sialylated structures (e.g., the previously proposed sialylated Hex_4_HexNAc_3-4_NeuAc compositions which as pyridylaminated glycans would be detected as *m/z* 1645 and 1848) were detected in any fraction. The superior separation of the glycans from contaminants as well as of isomers on the Kinetex column enabled us to confidently assign 100 structures, including glycans not found in the other HPLC experiments using an RP-amide column (Fig. 6; for MS/MS data for 58 assigned structures from this HPLC experiment, refer to Supplementary Fig. 5). In total, considering all analyzed *Drosophila* pools, some 120 anionic N-glycans can be proposed (Supplementary Table), including over 60 sulphated structures and nearly 40 PE-modified N-glycans, whereby some of these carry more than one anionic/zwitterionic modification.

### 3.3 Lectin reactivity to Drosophila N-glycans

Lectins and anti-carbohydrate antibodies are often used for rapid screens of glycosylation. In some initial experiments, Western blotting with *Sambucus nigra* and *Erythrina cristagalli* agglutinins was performed and showed an intense reaction with SNA, which was not abolished by sialidase or glucuronidase treatment (data not shown). This curious observation was followed up by preparation of a prototype fly N-glycan array using *Drosophila* Canton S and *nac1* adult flies, the latter having a greatly reduced content of fucosylated glycans [39,40], resulting in three pools (Canton S neutral, *nac* [1] neutral and a combined anionic pool). Glycans were labelled with AEAB and subject to 2D-HPLC, repooled based on epitopes defined by MALDI-TOF MS and normalized in terms of fluorescence intensity. The AEAB-labelled glycomes contained the same features as the Tf24 PA-labelled glycome and the anionic pool contained various glucuronylated, sulphated and phosphoethanolamine-modified N-glycans, which verifies that these features are found in different genetic backgrounds (Supplementary Fig. 6).

**Fig. 7:**
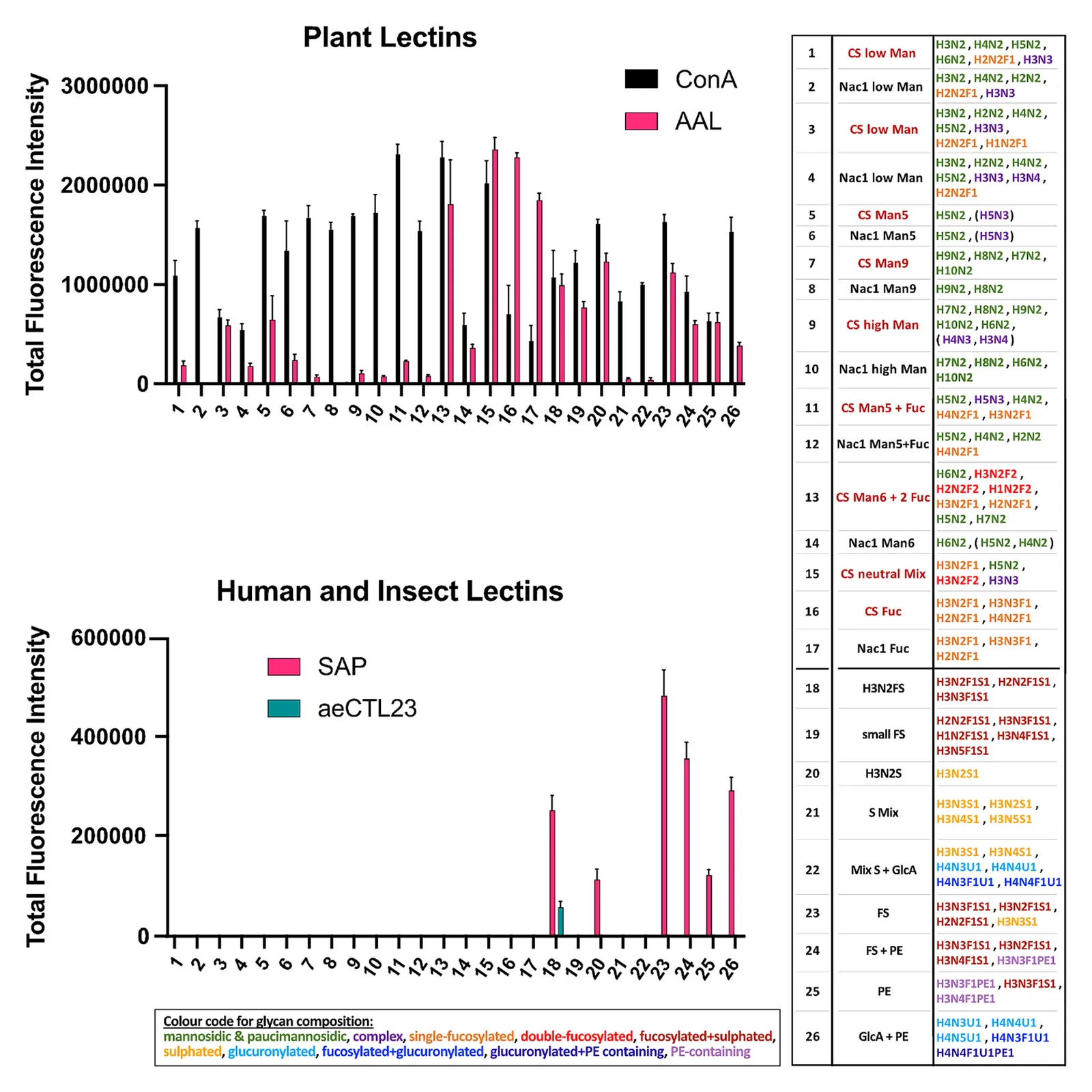
Lectin binding to a prototype Drosophila natural N-glycan microarray. Total fluorescence intensity values of the plant lectins concanavalin A (ConA) and *Aleuria aurantia* lectin (AAL) as well as the human pentraxin serum amyloid protein (SAP) and *Aedes aegyptii* aeCTL23 (insect lectin) respectively are shown with standard deviation after background subtraction. No binding signals were obtained for *Sambucus nigra, Maackia amurensis* and wheat germ agglutinins or three other *Aedes* lectins. The predominant epitopes are displayed on the x-Axis, while glycan compositions of the neutral pools of Canton S (abbreviated as CS) and *nac1* strains are shown on the right side; for further data regarding the glycan pools, refer to Supplementary Fig. 6.

The resulting prototype array was probed with different lectins and one pentraxin (Fig. 7); concanavalin A bound all fractions and *Aleuria aurantia* lectin only those shown by MALDI-TOF MS to contain mainly fucosylated structures, correlating also with the low levels of fucosylation in the *nac* mutant as compared to the Canton S wild type [40]. While human serum amyloid P (pentraxin) bound six of the nine anionic fractions, including all those with phosphoethanolamine-modified structures as well as fractions with sulphated forms of fucosylated paucimannosidic glycans, a mosquito lectin (aeCTL23, also known as mosGCTL-11) apparently bound a fraction containing glycans with sulphated fucose, no reactivity to *Sambucus nigra* agglutin was detected even in the anionic pool.

### 3.4 Activities of recombinant N-acetylglucosaminyltransferases II and IV

**Fig. 8:**
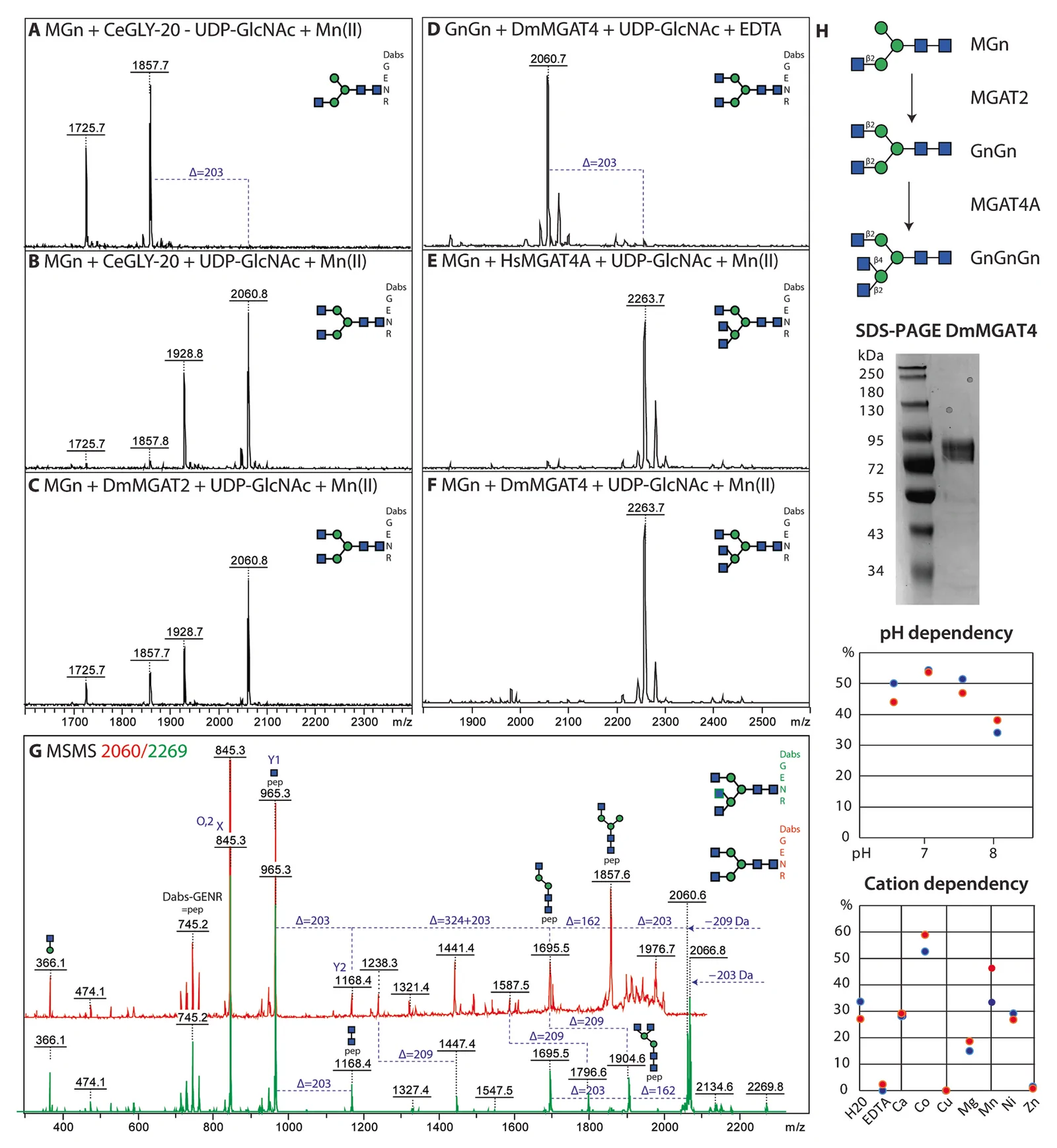
Activity of Drosophila *N*-acetylglucosaminyltransferases II and IV. (A-C) MALDI-TOF MS-based assays of *N*-acetylglucosaminyltransferase II activity with a dabsylated MGn glycopeptide with either *Caenorhabditis elegans* MGAT2/GLY-20 (expressed in HighFive cells) incubated in the absence or presence of UDP-GlcNAc or *Drosophila melanogaster* MGAT2 (p63 isoform expressed in HighFive cells) incubated in the presence of UDP-GlcNAc; the loss of 132 Da from substrate or product was due to laser-induced loss of part of the dabsyl moiety on the Ultraflex instrument. (D–F) MALDI-TOF MS-based assays of *N*-acetylglucosaminyltransferase IV activity with a dabsylated GnGn glycopeptide with either *D. melanogaster* MGAT4 (expressed in HEK293 cells) incubated in the presence of UDP-GlcNAc and EDTA or Mn(II) ions or *Homo sapiens* MGAT4A (commercial). (G) MALDI-TOF MS/MS of the GnGn substrate and GnGnGn MGAT4 product when using UDP-[U-^13^C]-GlcNAc (*m/z* 2060 and 2269); increments of 203 Da for GlcNAc or 209 Da for ^13^C-GlcNAc and the loss of the 1238 and 1587 fragment ions correlate with addition of a third antennal GlcNAc residue to the α1,3-mannose residue; it is assumed that the GlcNAc attached to the α1,6-mannose is more readily lost, than the β1,4-[^13^C]-GlcNAc attached to the α1,6-mannose. (H) Reactions catalysed by MGAT2 and MGAT4 and results of SDS-PAGE (0.5 μg), pH and cation dependency of purified Drosophila MGAT4; the predicted seven N-glycosylation sites are the probable reason for the wide molecular mass range of the protein (theoretically 66 kDa; see Supplementary Fig. 7).

Two alternatively spliced forms, p63 and p91 [36], of the *Drosophila* MGAT2 open reading frame have been described (CG7921) and these as well as both *Drosophila* MGAT4 isoforms (CG9384 and the neighbouring intronless CG17173) were transfected into *Pichia pastoris*; only the ‘short’ p63 MGAT2 showed any activity under the employed conditions (data not shown). Due to the low MGAT2 and no MGAT4 activities, alternative expression systems were sought. For MGAT2, we successfully detected activity and expression of a secreted form in HighFive cells, whereby a mono-antennary dabsylated glycopeptide with *m/z* 1857 was converted to one with *m/z* 2060 (Fig. 8A-C); expression in Sf9 cells with a C-terminal tag did not result in expression of active enzyme. In the case of MGAT4, we chose to pursue experiments with the CG9384 isoform phylogenetically closest to the single isoform in mosquitoes (Supplementary Fig. 7); expression of this enzyme in soluble form in HEK cells resulted in detection of an activity converting a biantennary dabsylated glycopeptide with *m/z* 2060 to one with *m/z* 2263 or, if using uniformly-^13^C-labelled GlcNAc, *m/z* 2269 (Fig. 8D-G). MGAT4 activity was abolished in the presence of EDTA, Cu(II) or Zn(II), but was highest in the presence of exogenous Co(II) or Mn(II) at pH 7 (Fig. 8H); a pseudohybrid glycan with only a β1,2-GlcNAc on the α1,6-mannose was not a substrate (data not shown). Combined with the data from Harry Schachter regarding recombinant *Drosophila* MGAT1 [41], we thereby show that the fruit fly has the enzymatic capacity to synthesis triantennary N-glycans of the types observed in the glycomic analyses.

## 4 Discussion

In comparison to previous studies by us and others, we demonstrate an expanded range of N-glycans in *Drosophila melanogaster*. In total, we propose more than 180 N-glycan structures (see Supplementary Table), which is within the range typical for our off-line HPLC-MALDI-TOF-MS studies on various invertebrates, around 120 of which are in the anionic fractions. Many of the older studies on fly glycans or glycoproteomes have yielded lists of 10–20 neutral masses or structures; 20 years ago, core difucosylation was determined to be the probable origin for the cross-reactivity of *Drosophila* glycoproteins with antisera recognising plant glycoproteins [9]. In 2007, anionic N-glycans in *Drosophila* were first proposed by Tiemeyer on the basis of permethylation data for embryos of the OreR strain, specifically sialylated and glucuronylated structures [11,12]; also, glucuronylation of N-glycans was observed upon knock-out of the FDL hexosaminidase in *Drosophila* S2 cells [42]. Glucuronylation of O-GalNAc and O-Fuc glycans as well as glycosphingolipids from *Drosophila* has also been determined [15,43]; for example structures, refer to Supplementary Fig. 8. Using a permethylation protocol optimised for sulphoglycomics, sulphation of mannose and core fucose on *Drosophila* N-glycans as well as on HexNAc residues of O-glycans was shown [16]. Sulphation and/or glucuronylation of N-glycans has been observed in other insects as well [16,31,37].

Here, we demonstrate that phosphoethanolamine is a zwitterionic modification of antennal HexNAc residues of *Drosophila* N-glycans, a decoration previously found on glycosphingolipids and O-glycans from this species [17,43] as well as on N-glycans from the honeybee [30,44], O-glycans from mosquitoes and wasps [16,45] and glycosphingolipids of *Drosophila* and other diptera [43,46]. Most of the detected phosphoethanolamine-modified N-glycans were also core fucosylated and glucuronylated. Indeed, in terms of absolute numbers, most anionic glycans were glucuronylated, including some triantennary structures, but the most intense peaks corresponded to sulphated structures. As in mosquitoes [16], sulphation was not only detected on paucimannosidic and hybrid/complex glycans, but also on many of the glucuronylated forms; in addition, some structures contained all three anionic/zwitterionic modifications, i.e., sulphate, glucuronic acid and phosphoethanolamine. In total, 97 glucuronylated structures, 67 sulphated structures and 38 PE-modified structures are proposed, which is a probable underestimate, as some further glycan masses even over 3000 Da were detected, but for which no interpretable MS/MS was obtained. The multiplicity of some isomers (e.g., two or three forms of most sulphated and glucuronylated structures) as well as the occurrence of isobars (e.g., *m/z* 1799 can be Man_8_GlcNAc_2_ or Man_3_-GlcNAc_3_Fuc_1_Gal_1_GlcA_1_PE) was resolved by MS/MS and chemical or enzymatic treatments. Interestingly, in terms of the prototype glycan array, the *Aedes* aeCTL23 lectin bound one fraction containing structures with sulphated core fucose. This lectin also recognises Rift Valley virus derived from insect cells as well as yeast polysaccharides [35]; however, further exploration of this result requires chemical synthesis of relevant ligands.

Unlike mosquitoes, the honeybee or lepidopteran species [16,30,31,44,47], no glycans with antennal fucosylation were detected, which also rules out the presence of XylGlcA-substituted antennal fucose as in Sf9 cells [47]. Finally, the occurrence of phosphoethanolamine on N-glycans in *Drosophila* contrasts with the phosphorylcholine found in lepidopteran species [47]. As part of screening a first-ever *Drosophila* N-glycan array, fractions containing phosphoethanolamine- and sulphate-modified glycans were recognised by human serum amyloid P (Fig. 7), a pentraxin previous described to bind phosphoethanolamine [48] as well as heparan sulphate [49]. In terms of comparing body and head samples, more difucosylated and more PE-modified glycans were detected in the head, suggesting an enrichment in neural tissue; certainly, the core α1,3-fucose epitope recognised by anti-horseradish peroxidase has been previously associated with neural and testes tissue in Drosophila [50,51].

Sialylation in insects has long been a controversial question, also in the context of expression of recombinant glycoproteins. As mentioned above, sialylated N-glycans (<0.1% of the total; Hex_4_HexNAc_3-4_NeuAc) were proposed on the basis of permethylation data in a glycomic analysis of trypsinised embryo powder of the OreR strain, whereby Y ions (the neutral loss of permethylated sialic acid from the parent ion) were detected, but no B ions (sialylated fragments) [12]. Since then, variations in amounts of sialylated N-glycans between heads of OreR and *cog7* mutant adults as well as a lack in third instar larvae brains of sialylation mutants have been reported [52–54]. Furthermore, insect α2,6-sialyl-transferases and CMP-sialic acid synthases have been shown to display enzymatic activity in vitro and/or biological functionality, but are expressed in different cell types [38,53,55,56]. However, we did not detect masses corresponding to sialylated N-glycans in our adult head sample, despite using the same workflow successfully used for analysing 2-aminopyridine-labelled sialylated echinoderm N-glycans [26]; conceivably this result may be due to the strain or stage of *Drosophila* used (Tf24 rather than OreR) or to instrument sensitivity, but is compatible with the lack of sialylation detectable by mass spectrometric techniques in other insects. The reactivity of fly proteins in blotting (data not shown) and histochemical experiments [57] towards *Sambucus nigra* agglutinin, generally accepted as being an α2,6-sialylation-specific lectin [58], thereby remains unexplained, also considering the lack of reactivity by this lectin in our glycan array experiment and a previous report that insufficient SNA-binding glycopeptides could be isolated from *Drosophila* heads for proteomic analysis [54].

The enzymatic origin of some of the N-glycan modifications has been elucidated and here we demonstrate also the activity of recombinant forms of GT16 and GT54 *N*-acetylglucosaminyltransferases II and IV (MGAT2 and MGAT4), the key enzymes for branching of N-glycans. Previously, the activity of Sf9 MGAT2 has been demonstrated [59], but, while the C-terminal lectin domain of *Bombyx* MGAT4 has been crystallised [60], no proof of an enzymatic function for any insect MGAT4 has been shown [61]. Insect MGAT4 sequences fall within the MGAT4A/B/D GT54 subfamily, which would correlate with it modifying the α1,3-mannose, rather than the α1,6-mannose [62]. There is no evidence for an MGAT5, thus earlier annotations of *Drosophila* triantennary N-glycans with a β1,6-antenna are incorrect [63], while the activity of insect MGAT3 homologues is also unproven. On the other hand, some processing α-mannosidases, *N*-acetylglucosaminyltransferase I, the core α1,3- and α1,6-fucosyltransferases and the FDL processing hexosamini-dases have been characterised in recombinant form [9,41,64–66]. Of the GT10 glycosyltransferase family members other than FucTA, no activity has yet been shown for *Drosophila* FucTB, FucTC and FucTD [9], in contrast to the Lewis-like activity of mosquito and honeybee FucTC [67,68]. Interestingly, FucTB is phylogenetically related to human FUT10 and FUT11, which were recently shown to be peptide O-fucosyltransferases [69]; FucTC in the standard Drosophila Canton S strain has a premature stop codon, unlike the sequence in the Tf24 strain analyzed in the current study. On the basis of the proven enzymatic functions and the N-glycomic analyses, it can be concluded that the N-glycan fucosylation potential of *Drosophila* is apparently reduced compared to other insects.

Use of specific galactosidases on insect glycans, including the β1,3-galactosidase employed here [70], indicate that *D. melanogaster* Tf24 adults do not synthesise typical LacNAc antennae, but rather the type I isomer with β1,3-linked galactose, which contrasts previous annotations for OreR and Canton S embryos [12,63]. Most of the galactosylated antennae in Tf24 adults are glucuronylated and, unlike a previous study on OreR embryos [11], we do not propose ‘naked’ LacdiNAc antennae, but rather mainly GlcAβ1,3Gal-capped forms with or without phosphoethanolamine. Both our observations indicate that when ‘complex’ glycan modifications are initiated, then they tend to go to completion, resulting in low levels of the neutral intermediates. This may be due to the accessibility of the glycans to processing enzymes in the Golgi: if the glycans are destined for secretion or the cell surface and are not buried within the glycoprotein structure, they are either processed ‘down’ to paucimannosidic forms or processed ‘up’ to more complicated antennae. Regarding the proven antennae, one GT31 LacdiNAc-modifying β1,3-galactosyltransferase (CG33145), one GT7 LacdiNAc-forming β1,4-*N*-acetylgalactosaminyltransferase (β4GalNAcTA; CG8536) and two GT43 β1,3-glucuronyltransferases (GlcAT-S and -P, otherwise known as GlcAT-BSI and BSII; CG3881 and CG6207) have been shown to have activity towards N-glycans in vitro [71–73], but their role in fly glycan biosynthesis in vivo has not been demonstrated. Other ‘unknowns’ pertain to the GlcNAc-modifying β1,3-galactosyltransferase, fucose and mannose-modifying sulphotransferases and HexNAc-modifying phosphoethanolaminyltransferases required to modify N-, O- and lipid-linked glycans. This relative lack of knowledge impacts our ability to determine the in vivo function of all these modifications. On the other hand, the basics of *Drosophila* N-glycan biosynthesis (for a potential biosynthetic scheme, refer to Supplementary Fig. 8) do follow a GO/NOGO ‘yellow brick road’ of the type postulated by Harry Schachter for mammals [74], whereby the control of branching via bisecting MGAT3 is absent in insects, but a hexosaminidase in the insect Golgi determines the high occurrence of paucimannosidic structures.

In terms of biology, the phenotypes resulting from mutations in genes required for Golgi processing of N-glycans in *Drosophila* indicate that glycan structures affect neuronal function, lifespan and innate immunity. Here the contribution of Harry Schachter, his collaborators and others yielded important insights [75–78]: interestingly, mutations of the *mgat1* gene resulted in a fused lobes phenotype also seen for mutations affecting the FDL Golgi hexosaminidase [66], which suggests an important role for the dominant paucimannosidic glycans in neural development. However, these studies were not extended to *mgat2* or *mgat4*, but mutations in the human MGAT2 gene are also associated with neurological defects [79]. On the other hand, ectopic expression of the core α1,3-fucosyltransferase FucTA also impacts the neuronal system perhaps by diversion of GDP-Fuc from the O-fucosylation pathway [80], indicative of the fine glyco-metabolic balances in vivo. More global effects on N-glycosylation are due to mutations of the ER glucosidase GCS1, which affect self-tolerance [81]. However, such data do not show the specific importance of sulphation, glucuronylation or phosphoethanolamine modification of *Drosophila* N-glycans, in contrast to our knowledge regarding the impact of the first two mediated via glycosaminoglycans and O-glycans [82]. As stated above, ascertaining the roles of the anionic and zwitterionic modifications of fly N-glycans requires the identification of the relevant transferases and of lectin receptors; however, the occurrence of such moieties in a variety of insects is an indication of selection pressure during evolution resulting in their emergence, species-specificity and retention, which itself shows their biological significance.

## Supplementary Material

Supplementary data to this article can be found online at https://doi.org/10.1016/j.bbagen.2026.130961.

Supplement

## Data Availability

Selected mzxml files are available via Glycopost, GPST000677: https://glycopost.glycosmos.org/entry/GPST000677.
