# Supplementary material for: Complex anionic and zwitterionic N-glycans in Drosophila melanogaster
Source: Biochim Biophys Acta Gen Subj. Author manuscript; Available in PMC 2026 Oct 16. (PMC7619574; doi:10.1016/j.bbagen.2026.130961)
Supplement: Supplementary file 1 [file EMS220408-supplement-Supplement.pdf]

Complex anionic and zwitterionic N-glycans in *Drosophila melanogaster*

Kristína Maďarová, Zuzanna Dutkiewicz, Lena Nuschy, Maria Magdalena John,
Huijie Wang (王彗捷), Barbara Eckmair, Iain B. H. Wilson and Katharina Paschinger

Institut für Biochemie, Universität für Bodenkultur, 1190 Wien, Austria

Correspondence should be addressed to: iain.wilson@boku.ac.at

Supplement

Further information regarding the glycomic analyses 2

Further information regarding the glycan array analyses 4

Supplementary Figures 1-8 6

Supplementary Table 16

Further information regarding the glycomic analyses

Definition of the level of the glycan structural analysis:

The goal was the N-glycomic analysis of *Drosophila melanogaster* wild-type Tf24 strain - heads, bodies and whole adult flies. Thereby, either individual glycan-containing HPLC fractions or whole N-glycomes were subject to MALDI-TOF MS and MS/MS.

Search parameters and acceptance criteria:

a. **Peak lists:** As stated in the methods section: typically, 2000-4000 shots were summed for MALDI-TOF MS and 10000-20000 for MS/MS. Spectra were processed with the manufacturer's software (Bruker Flexanalysis 3.3.80) using the SNAP algorithm with a signal/noise threshold of 6 for MS (unsmoothed) and 3 for MS/MS (four-times smoothed).
b. **Search engine, database and fixed modifications:** All glycan data were manually interpreted and no search engine or database was employed; the fixed modification is the 2-aminopyridine label at the reducing end ($GlcNAc_1$-PA fragments of *m/z* 300).
c. **Exclusion of known contaminants and threshold:** All glycan data were manually interpreted; only peaks with an MS/MS consistent with a pyridylaminated chitobiose core were included – the 'threshold' for inclusion was an interpretable MS/MS spectrum (at least in terms of composition).
d. **Enzyme specificity:** A description of the PNGase F and A release method is given in the methods section; the latter enzyme removes N-glycans from glycopeptides regardless of the presence of core α1,3-fucose on the reducing-terminal GlcNAc. Exoglycosidase digestions were performed on selected glycan fractions to determine linkages of some glycan motifs.
e. **Isobaric/isomeric assignments:** For isomeric species, differences in RP-HPLC elution and MS/MS were used for the assignment (as described in the text).

Glycan or glycoconjugate identification:

a. **Precursor charge and mass/charge (*m/z*):** All glycans detected were singly-charged. For the positive mode, the *m/z* values are for protonated forms. Depending on the glycan amount or preparation, the relative amounts of the H^+, Na^+ and K^+ adducts varied. Maximally two decimal places used for the *m/z* annotations consistent with the accuracy of MALDI-TOF MS; in the figures and due to space limitations, only one decimal place is presented. Previous data indicate an average +0.03 Da (+22 ppm) deviation between the measured and the calculated *m/z* values on the instrument used.
b. **MALDI-TOF MS settings (positive mode):** For Autoflex Speed: Ion Source 1 and 2 were 19.00 and 16.75 kV; Lens, 9.00 kV; Reflector 1 and 2, 21.05 and 9.65 kV; Pulsed Ion Extraction, 160 ns; Matrix Suppression typically up to 700 Da; Detector Gain, typically 2163 V. For Rapiflex: reflector voltage, lens voltage, and gain of 20.8 kV, 11.6 kV, and 1909 V respectively.
c. **MALDI-TOF MS/MS settings (positive mode):** For Autoflex Speed: Ion Source 1 and 2 were 6.00 and 5.35 kV; Lens, 2.90 kV; Reflector 1 and 2, 27.00 and 11.75 kV; Lift 1 and 2, 19.00 and 4.00 kV; Pulsed Ion Extraction, 140 ns; Detector Gain, typically 2260 V when fragmenting; Laser Power Boost typically 50%; not in CID mode; PCIS typically 0.65%. For Rapiflex: reflector voltage, lift voltage, and gain of 23.8 kV, 19 kV, and 2171 V respectively; PCIS 0.3%.
d. **All assignments:** For the glycans present in each pool, see the RP-HPLC chromatograms annotated with structures shown according to the Standard Nomenclature for Glycans.
e. **Modifications observed:** Listed are the *m/z* values for glycans carrying a reducing terminal pyridylamine group as judged by presence of an *m/z* 300 $GlcNAc_1$-PA fragment. As the glycans are otherwise chemically unmodified, $\Delta m/z$ of 80, 123, 146, 162, 176 and 203 correspond to sulphate (detected in negative mode), phosphoethanolamine, fucose (deoxyhexose), hexose,

glucuronic acid (hexuronic acid; detected in positive and negative modes, therefore ruling out the isobaric methylhexose as a possibility) or *N*-acetylhexosamine. There was no indication for the presence of phosphate or sialic acid residues. While pyridylamine is a chemical modification of the N-glycans performed during the glycomic workflow, phosphoethanolamine is a natural glycomic modification found in some insect species on N-glycans, O-glycans and glycosphingolipids; phosphoethanolamine in mammals occurs as the headgroup of phosphatidylethanolamine lipids.

f. **Number of assigned masses:** Glycan assignments were not just based on measured mass only, but on the basis of MS/MS corroborated by elution data.

g. **Spectra:** Representative annotated spectra (MS and MS/MS) defining structural elements are given in various figures. In total, over 100 MS or MS/MS spectra reflecting the data for the approximately 180 defined structures are shown in the main text or supplement; 175 mzxml files have been uploaded to Glycopost (GPST000677).

h. **Structural assignments:** As noted in the results section, the typical oligomannosidic structures are assigned based on fragmentation pattern and elution time; as shown in previous studies, RP-HPLC retention is affected by the type of core fucosylation (core α1,6-fucosylation resulting in later retention), by the antennae (glycans with 'lower arm' α1,3-antennae elute earlier than those with 'upper arm' α1,6-antennae; partly verified by specific mannosidase digestions) and by sulphation (earlier retention if on α1,6-mannose, later if on α1,6-fucose). It is otherwise assumed that the glycans contain a di- or tri-mannosyl core consistent with typical eukaryotic N-glycan biosynthesis and that there is processing by GlcNAc-transferases and core α1,3/6-fucosyltransferases as in a range of multicellular organisms. Antennal PE-modified LacdiNAc and type I lacto-motifs have been defined for honeybee N-glycans on the basis of hydrofluoric acid treatments (Hykollari et al., 2019) as also shown here. The glucuronylated antennae are assumed to be either GlcAβ1,3Galβ1,3GlcNAc or GlcAβ1,3Galβ1,3GalNAcβ1,4GlcNAc, as previously observed in mosquito samples (Kurz et al., 2015); these GlcA residues were shown to be β-glucuronidase-sensitive. The sulphated glycans are observed in negative mode as $[M-H]^-$ ions, but in-source loss results in the observation of $[M-SO_3]^+$ ions in positive mode - a property contrasting with phosphorylated glycans which would be observed in both modes.

Summary of glycosidases used in this study

PNGases	*Flavobacterium* PNGase F (recombinant, *E. coli*)<br>*Oryza sativa* PNGase A (recombinant, *Pichia*)
Fucosidase	Bovine α-fucosidase (native)
Galactosidase	*Xanthomonas manihotis* β1,3-galactosidase (recombinant, *E. coli*)
Glucuronidase	Human β-glucuronidase (recombinant, murine NS0 cells)
Hexosaminidase	Jack bean β-*N*-acetylhexosaminidase (native)
Mannosidases	Jack bean α-mannosidase (recombinant, *Pichia*)<br>*Xanthomonas manihotis* α1,6-mannosidase (recombinant, *E. coli*)

Further information regarding the glycan array analyses

**1. Glycan Binding Samples**	
Description of Sample	*Fungal/plant lectins:* Concanavalin A (ConA), *Aleuria aurantia* Lectin (AAL); wheat germ agglutinin (WGA), *Sambucus nigra* agglutinin (SNA), *Maackia amurensis* lectins (MAA/MAL-I and -II) (Vector Labs) used at 1:100 (20-50 µg/ml).<br>*Human pentraxin:* Human serum amyloid P (SAP; Fitzgerald).<br>*Insect C-type lectin:* Fc-tagged Aedes aeCTL23 (Prof. Lepenies, München).<br>All dilutions were in TSM (20 mM Tris-HCl, pH 7.4, 150 mM NaCl, 2 mM $CaCl_2$, 2 mM $MgCl_2$) supplemented with 0.05 % Tween-20, 5 mM $CaCl_2$, 1% bovine serum albumin (hereafter TSMBB).
Assay protocol	The slides were incubated (all dilutions in TSMBB) with either:<br>(i) Biotinylated forms of recombinant or commercial lectins (1:100) followed by AF647 conjugated streptavidin (Invitrogen, 2 µg/ml).<br>(ii) Human serum amyloid P (1:200) followed by anti-SAP IgG from mouse (Calbiochem; diluted 1:1000) and finally anti-rabbit IgG, AF 647 (Invitrogen, 2 µg/ml).<br>(iii) Fc-tagged *Aedes* aeCTL23 (1:100) followed by anti-human IgG (Biolegend; 1:100) and finally anti-mouse IgG, AF 647 (Invitrogen, 2 µg/ml).<br>Controls: AF647-conjugated streptavidin or secondary antibodies without prior application of lectins or pentraxin; in the case of SAP and aeCTL23, addition of 25 mM EDTA resulted in abolition of signal.
Incubation and washing	Each incubation step was one hour; washing was by dipping ten times in TSMWB (TSM with 0.05% Tween-20) and then water, followed by drying.
Sample modifications	Fluorescent antibodies (conjugated with AlexaFluor-647) were used which detect the relevant primary antibodies or which were specific for biotin in order to detect binding of biotinylated lectins.
**2. Glycan Library**	
Glycan description for defined glycans	Glycans are abbreviated based on their composition
Glycan description for undefined glycans	N-glycans were prepared by PNGaseF/A release of glycopeptides derived by pepsin proteolysis of *D. melanogaster* homogenates; see the experimental procedures for further details regarding isolation, AEAB-labelling and HPLC as well as Supplementary Figure 6 for HPLC profiles and selected MS/MS. Normalisation was on the basis of fluorescence intensity of HPLC peaks. Detailed analysis of PA-labelled *D. melanogaster* N-glycans are presented.
Glycan modifications	Glycan pools were derivatised with AEAB and the pools were HPLC purified to remove residual linker prior to re-pooling.
**3. Printing Surface**	
Description of Surface	NHS-ester activated glass slide
Manufacturer	SCHOTT (NEXTERION SlideH, 1070936)
Covalent Immobilization	Glycans are terminated with a primary amine linker

4. Arrayer (Printer)	
Description of Arrayer	Scienion Flexarrayer S1
Dispensing mechanism	Non-contact printing with the supplier's nozzles (Type 3) and a pulse of 45 µs, ca. 103 V.
Glycan deposition	Five replicates of 0.8 nl each; estimated as 2-4 µM as compared to a standard AEAB-labelled glycan.
Printing conditions	Derivatised glycans and oligosaccharides were mixed 1:1 with spotting buffer (150 mM sodium phosphate pH 8.5); printing was at room temperature; arrays were left to hybridise overnight prior to blocking (50 mM ethanolamine in 50 mM sodium borate, pH 9.0) for 1 h at RT, washing (in TSM + Tween and finally H_2O) and drying.
**5. Glycan Microarray**	
Array layout	14 subarrays per slide with 5 replicates per glycan sample.
Glycan identification and quality control	Glycans on the array were pools of natural glycans. All glycans were HPLC purified and verified by MALDI-TOF MS. Spotting buffer alone was used as one of the negative controls.
**6. Detector and Data Processing**	
Scanning hardware	GenePix 4400A Scanner
Scanner settings	Multiple photomultiplier tube (PMT) gain values from 800-1000 and 100% laser power; wavelength 635 nm.
Image analysis software	GenePix Pro 7.3
Data processing	Raw .tif image files were imported to GenePix 7.3.0.0; fluorescent intensities of spots in each sub-array were analysed. Resulting data were exported into Excel; raw fluorescent values were background subtracted and evaluated in Excel and GraphPad Prism.
**7. Glycan Microarray Data Presentation and Interpretation**	
Data presentation	Bar charts with error bars are shown in Figure 7. No software used for interpretation.

Supplementary Figure 1: Workflow and MALDI-TOF MS profiles for body and head samples.
Drosophila melanogaster Tf24 fly samples (whole fly, head or body) were processed according to the depicted workflow prior to derivatisation with 2-aminopyridine. The initial MALDI-TOF MS profiles for the body and head neutral and anionic samples show relatively simple profiles (pauci- or oligomannosidic for the neutral samples, a difucosylated structure in the head; sulphation and glucuronylation for the anionic samples, with some phosphoethanolamine modified glycans observed in the overall profile for the head).

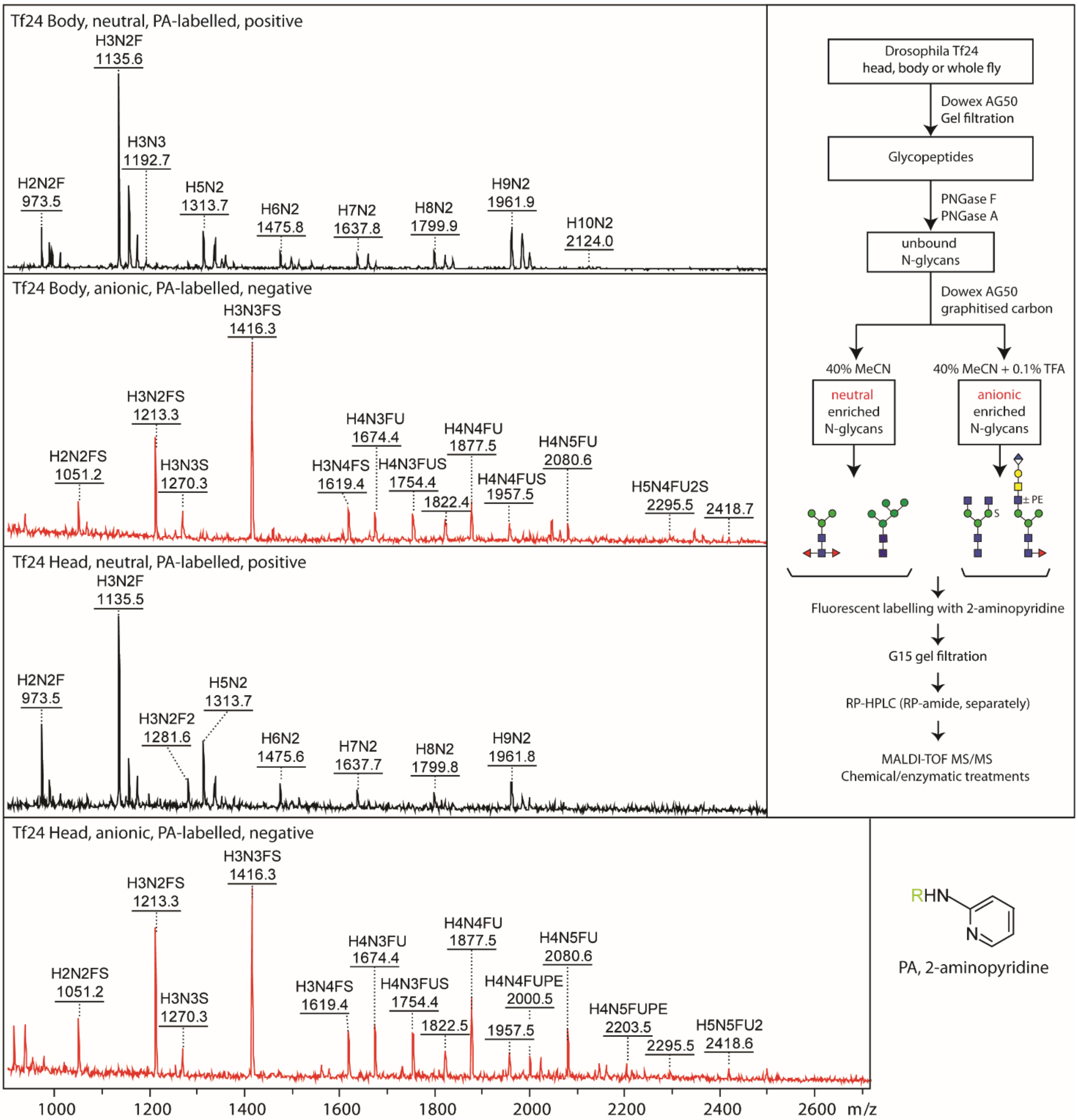

Supplementary Figure 2: Alternative workflow for glycomic analysis of Drosophila heads and analysis of glycans released by PNGase A. **(A)** Tf24 head glycopeptides were subject to PNGase F and PNGase A digestion, whereby PNGase F treatment was followed by a Dowex chromatography step and the released glycans separated from the remaining glycopeptides, which were then treated with PNGase A. **(B)** While the PNGase F-treated neutral and anionic glycans were not combined and the anionic glycans were fractionated on a Kinetex column (see ***Figure 6*** in the main text), the PNGase A-released neutral and anionic pools were combined and subject to HPLC on an RP-amide column; some residual mannosidic and solely α1,6-fucosylated glycans remained, but the major peak at 6.5 g.u. as well as all earlier-eluting peaks contained difucosylated structures, including some glucuronylated, sulphated and PE-modified glycans. **(C)** Selected MS/MS in positive and negative mode are shown for five difucosylated structures. The signature Y1 *m/z* 592 fragment is characterisitic for core difucosylation; PE-modified antennae are defined by neutral losses in positive mode, while a doubly PE-modified glycan showed a dominant *m/z* 813 B-ion fragment in negative mode. For MS/MS of the sulphated *m/z* 1562 glycan and the PE- and GlcA-modified difucosylated glycan with *m/z* 2148, refer respectively to ***Figures 3G and 5 K/L*** in the main text.

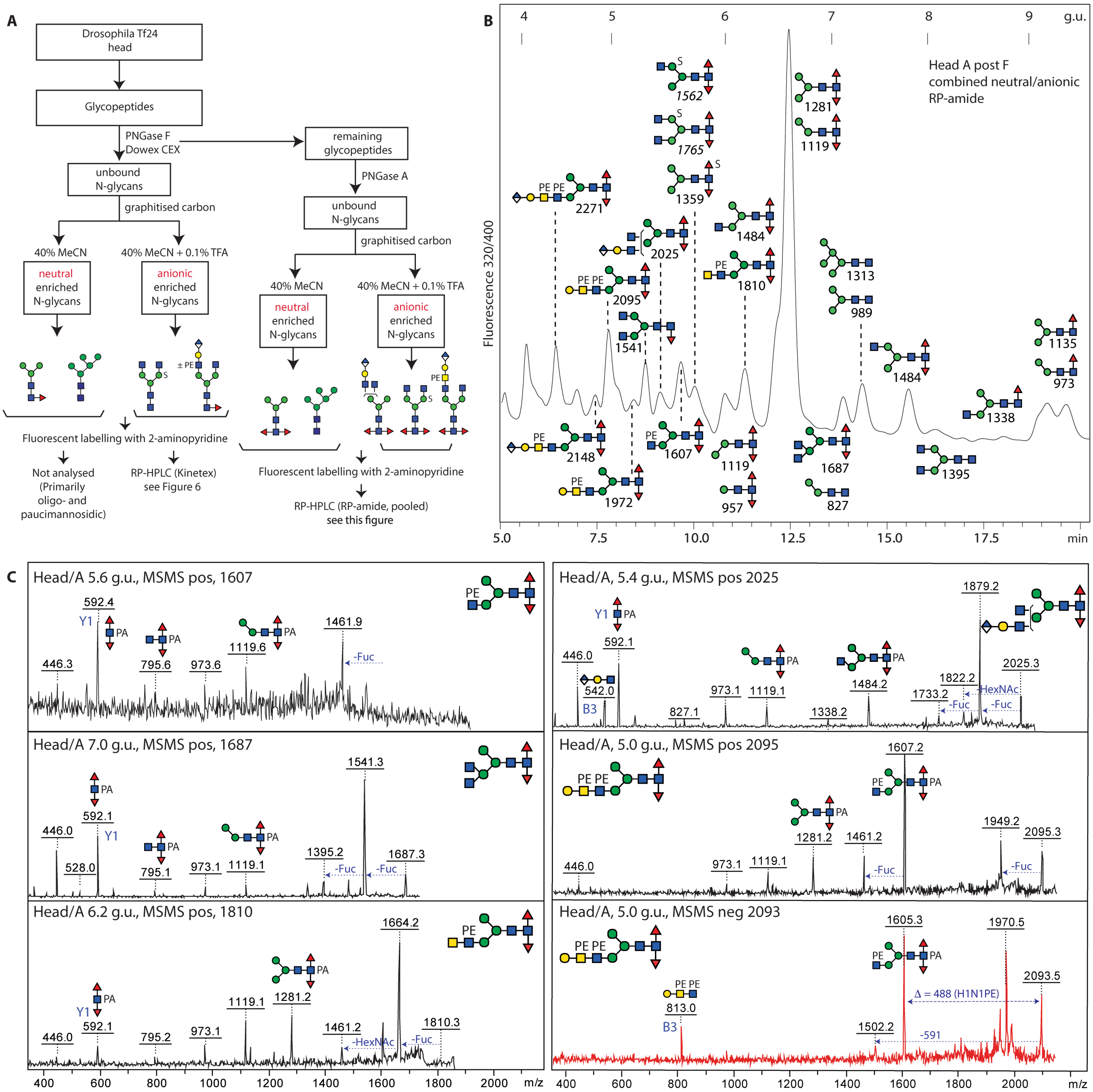

Supplementary Figure 3: HPLC N-glycomic profiles for whole adult flies. The RP-amide profiles for the PNGase F/PNGase A-released neutral and anionic N-glycans from whole adults can be compared to those for the body and head samples shown in the main text (***Figures 1 and 2***).

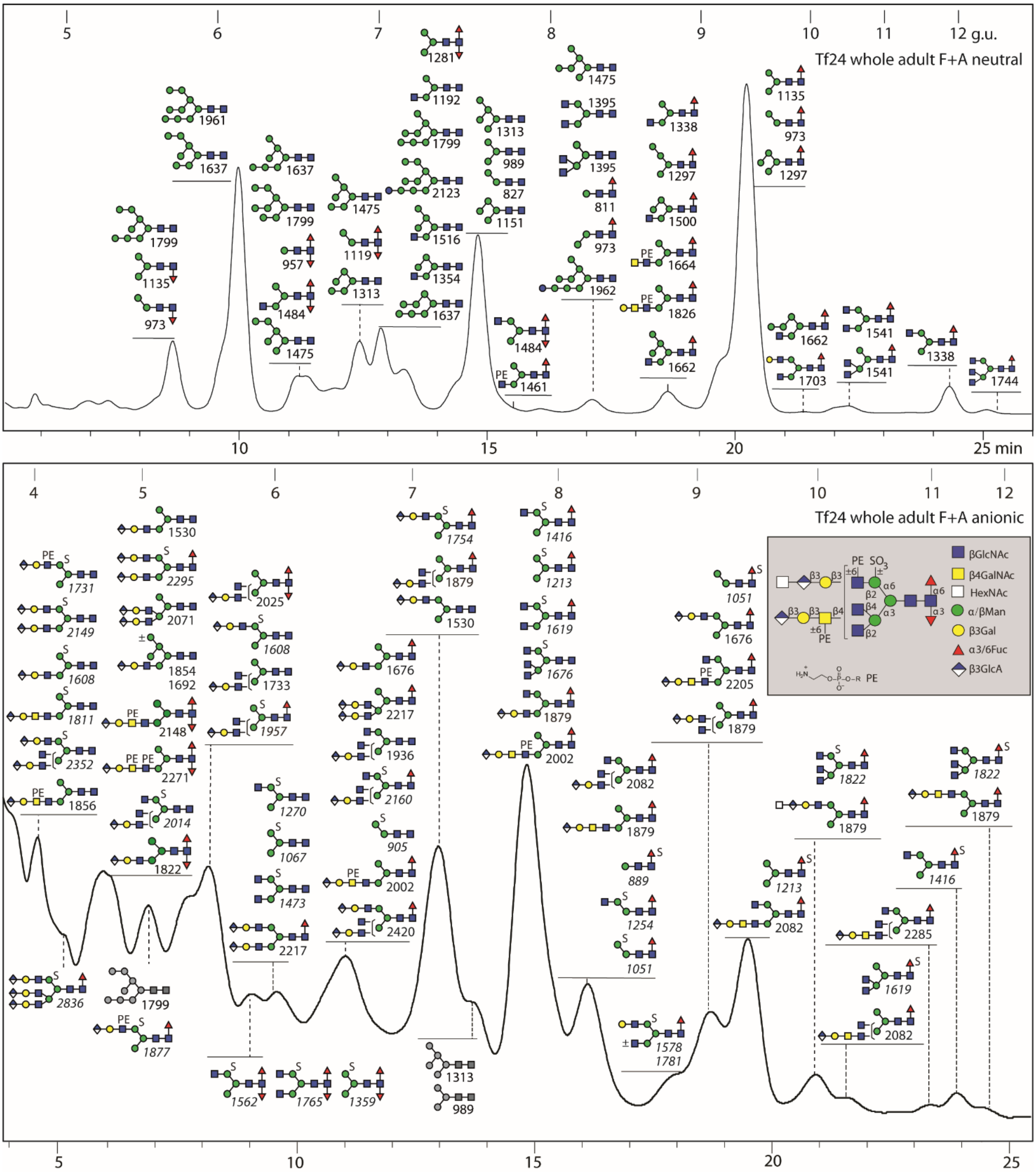

Supplementary Figure 4: MALDI-TOF MS/MS of Canton S fly glycans. N-glycans from a preparation fractionated in 2000 AD, i.e., about twenty-five years ago and stored at -20 °C since then (Fabini et al, *J. Biol. Chem.*, 2001), were reanalysed in 2025 by MALDI-TOF MS/MS showing that sulphated, glucuronylated and phosphoethanolamine-modified glycans were in these old preparations, but were then below the levels of detection; the MS/MS spectra show the basic characteristics for the same glycans from the new preparations. The isomeric status of the major difucosylated and two oligomannosidic glycans could be also verified.

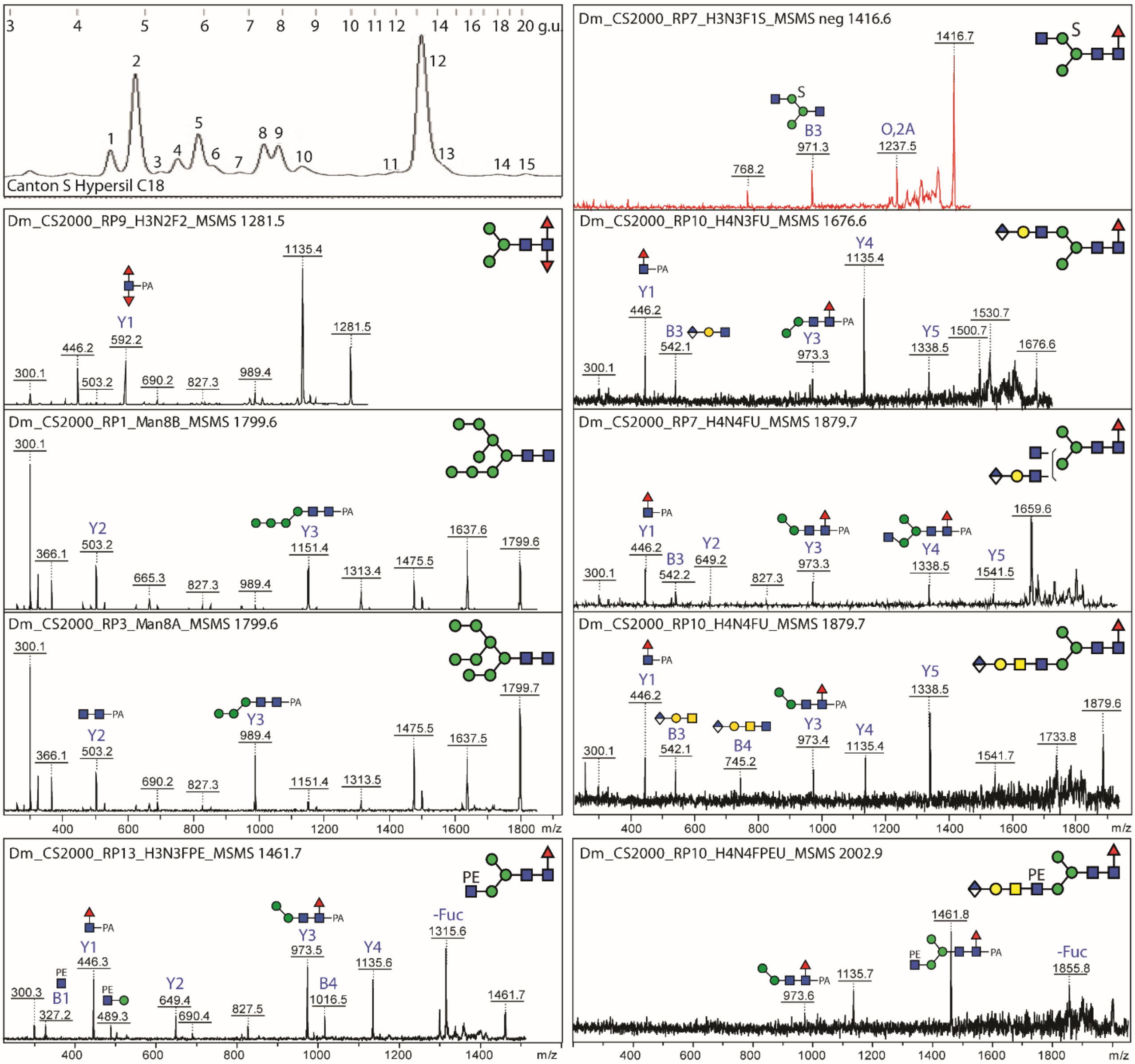

Supplementary Figure 5A. MALDI-TOF MS/MS of Kinetex fractions A-K of the PNGase F-released anionic subpool of Tf24 head N-glycans. Each Kinetex fraction (see ***Figure 6*** of the main text) was subject to MALDI-TOF MS/MS in positive (black) and negative (red) modes; in positive mode, sulphated glycans show in source loss of sulphate. Negative mode MS/MS spectra of glycans carrying both sulphate and glucuronic acid show neutral losses of 80 or 176 Da from the molecular ion, but loss of all anionic moieties results in a loss of negative mode signal, hence the lack of lower *m/z* fragments. Selected glycosidase and HF digestions were also performed and the effects are summarised in the headings: HF, hydrofluoric acid; β3Gal, β1,3-specific galactosidase; βGlcAase, β-glucuronidase; BovFuc, bovine α-fucosidase; JBHex, jack bean β-hexosaminidase; JBMan, jack bean α-mannosidase. Note that PE modifications or a disubstituted α1,3-mannose can apparently interfere with the action of the glucuronidase.

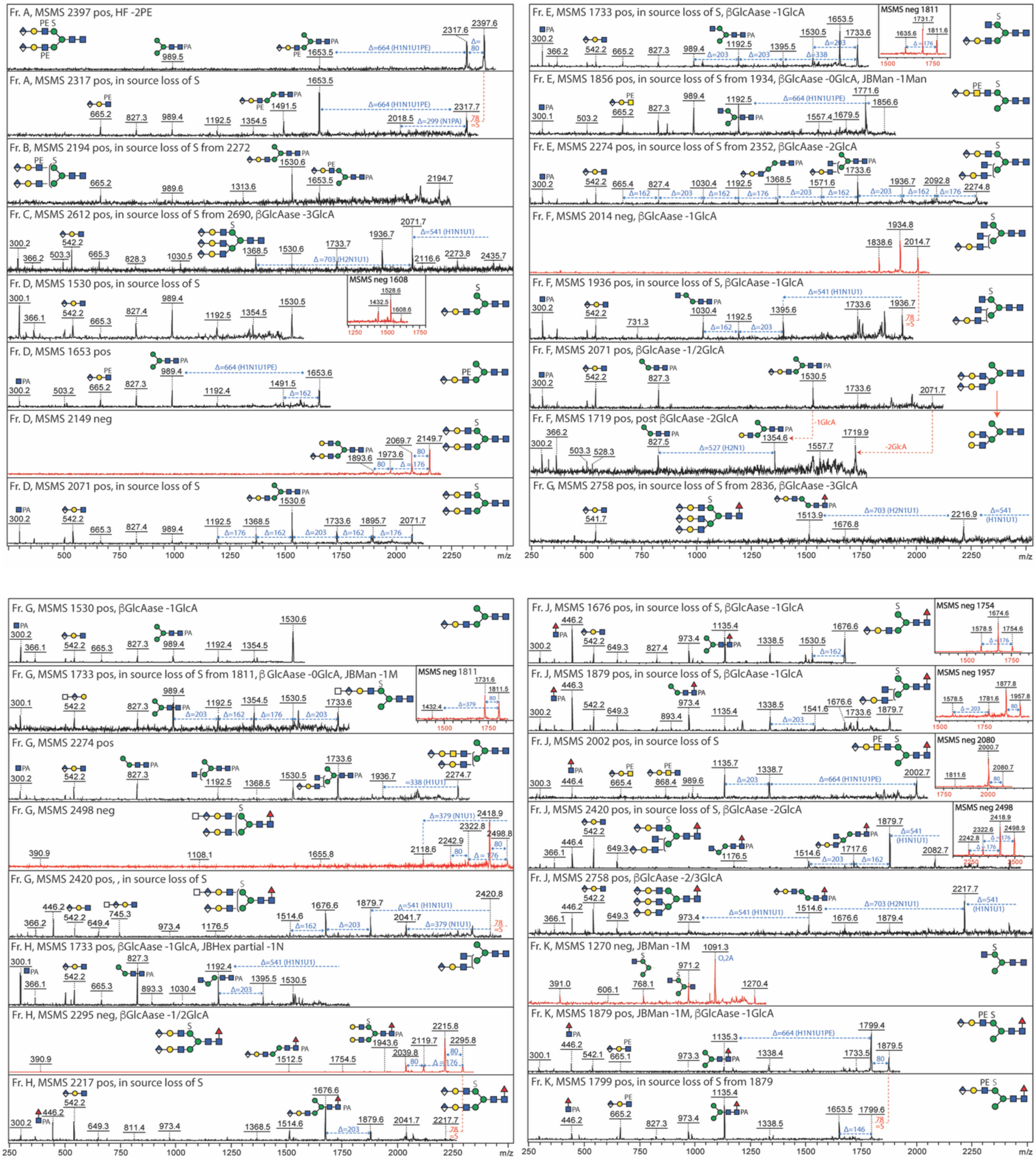

Supplementary Figure 5B. MALDI-TOF MS/MS of Kinetex fractions L-Y of the PNGase F-released anionic subpool of Tf24 head N-glycans. Each Kinetex fraction (see ***Figure 6*** of the main text) was subject to MALDI-TOF MS/MS in positive (black) and negative (red) modes. Selected glycosidase and HF digestions were performed (see notes on previous page). For 'upper arm' isomers, characteristics are complete removal of the α1,3-mannose by jack bean mannosidase and the late retention time. Sulphated glycans lacking glucuronylated antennae were detected in negative mode and isomers were distinguished by fragmentation pattern and mannosidase or fucosidase sensitivity/resistance; the underlying parent structure was observed in positive mode. The distinct MS/MS spectra for even closely-eluting isomers are indicative of different structures (e.g., isomers of *m/z* 1879, 2002, 2082, 2205 or 2420). For a comparison of the positive mode spectra for *m/z* 2125 post HF treatment as well as the corresponding negative mode spectrum (fraction S), refer to ***Figure 5H-J*** in the main text.

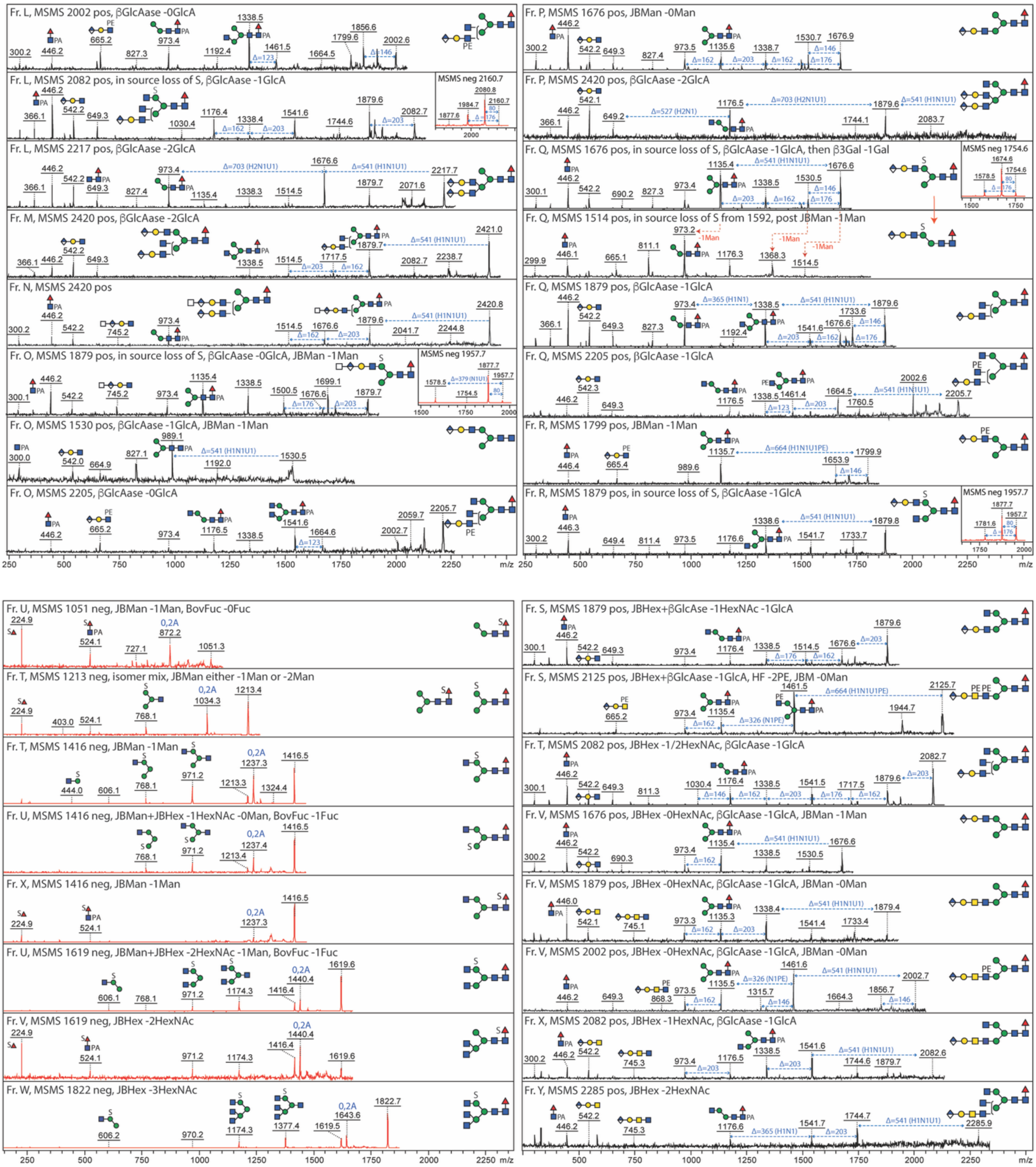

Supplementary Figure 6A: AEAB-labelled Drosophila glycans from Canton S (abbreviated as CS) and nac1 strains. Neutral (40% ACN elution) and anionic (40% ACN + 0.1% TFA elution) glycans were labelled with AEAB separately and purified by NP-HPLC (Zorbax column). Fractions were pooled (retention time is indicated) and further separated by RP-HPLC (Kinetex column), the respective chromatograms are shown. Fractions were combined for the glycan microarray based on MALDI-TOF MS and MS/MS analysis, regions are highlighted in color in the chromatograms. Fluorescent intensities of glycan pools were normalised in order to print equal amounts (estimated concentrations of 2-4 µM as compared to a standard). The glycan composition of the pools is indicated; selected MS/MS are shown on the next page.

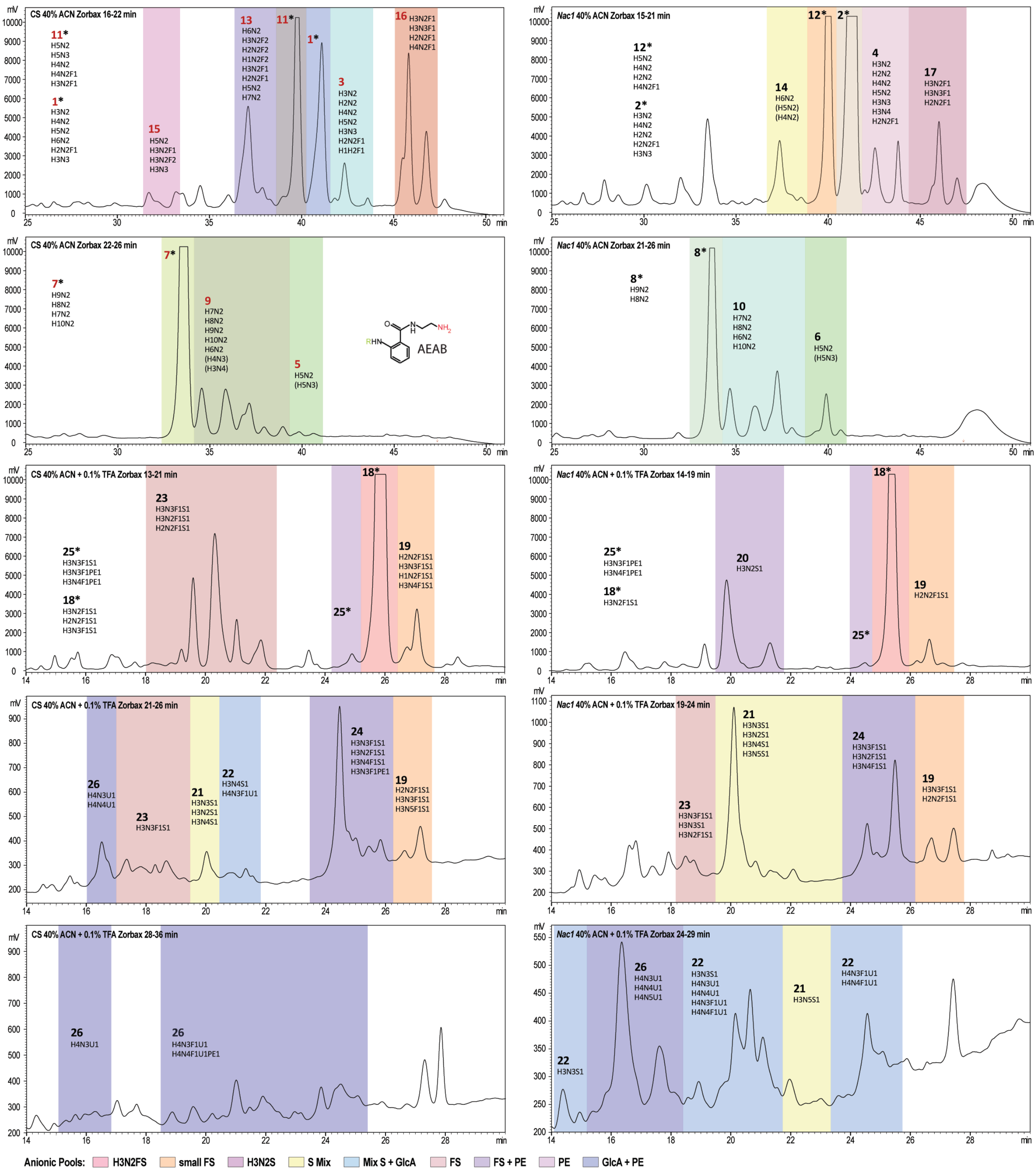

Supplementary Figure 6B: AEAB-labelled Drosophila glycans from Canton S (abbreviated as CS) and nac1 strains. Selected MS/MS of AEAB-labelled glycans in either positive or negative mode (designated NEG). As the difference in mass between AEAB and PA-labelled glycans is 85 Da, B-ion fragments are the same for both classes of glycan, but Y-ion fragments differ by 85 a.m.u. Each spectrum is annotated with the relevant normal phase pool name and reversed phase fraction number, the composition, the *m/z* and the pool designation as shown in ***Figure 7*** in the main text. **(i-iii)** MALDI-TOF MS/MS of the three neutral paucimannosidic glycans differ in terms of mono- or difucosylation of the core GlcNAc. **(iv-vi)** The three neutral oligomannosidic glycans differ in terms of the $Man_{5\text{-}7}GlcNAc_1$ B ions and the $Man_{2\text{-}3}GlcNAc_2$-AEAB Y3 ions. **(vii)** The simple biantennary glycan displays serial losses of HexNAc or Hex residues. **(viii-xii)** MS/MS of the five sulphated glycans, which all show an $^{O,2}A$ cross-ring cleavage, differ in terms of the intensities of ions at, e.g., *m/z* 606, 609 and 768; by comparison to the properties of PA-labelled glycans, late retention times and an *m/z* 609 fragment are proposed to be associated with sulphation of fucose and earlier retention times and the presence and/or higher intensity of *m/z* 606, 768 and 971 ions are proposed to be associated with sulphation of mannose. Co-fragmentations due to other co-eluting glycans or sulphate migration can explain the inconsistent low intensity ions. **(xiii)** MS/MS of the example PE-modified glycan shows B-ions at *m/z* 327, 489 and 1016 proposed to correspond to $Hex_{0\text{-}1}HexNAc_1PE$ or $Hex_3HexNAc_2PE$ and a Y ion at 1058 resulting from the loss of 488 a.m.u.. **(xiv)** MS/MS of the example glucuronylated glycan shows an $^{O,2}A$ cross-ring cleavage and a B3 ion proposed to correspond to $HexA_1Hex_1HexNAc_1$.

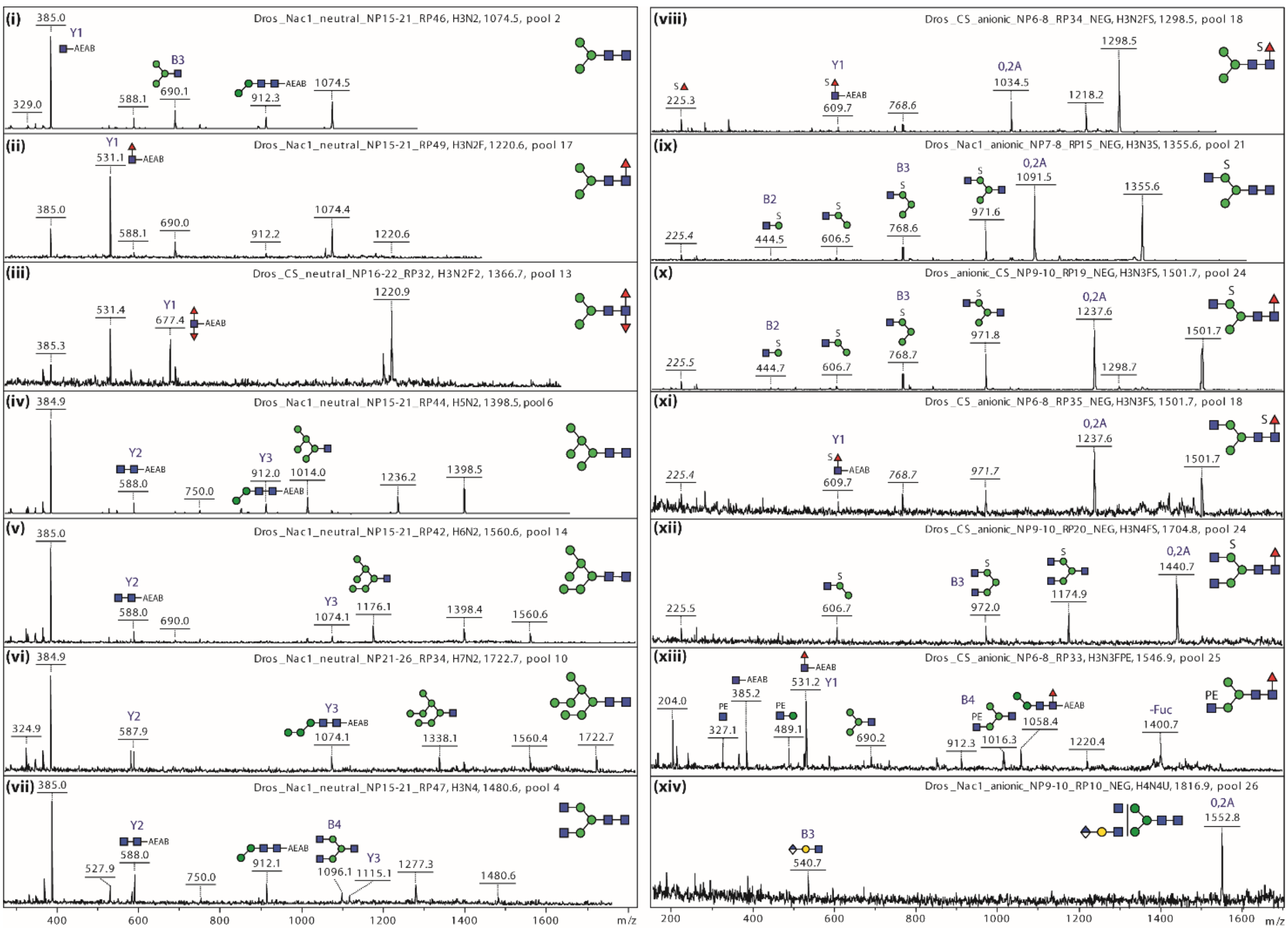

Supplementary Figure 7: Sequence analysis of D. melanogaster MGAT4. Insect and human *N*-acetylglucosaminyltransferase IV GT54 sequences were aligned using Multalin (multalin.toulouse.inra.fr); the resulting alignments and tree display indicate that *Drosophila* CG9384, shown to be enzymatically active (***Figure 8*** of main text), is closely related to other Dipteran MGAT4 sequences (*Aedes aegypti* and *Anopheles gambiae*), while *Drosophila* CG17173 (encoded by an intronless gene neighbouring CG9384) is more divergent, even in comparison to Lepidopteran MGAT4 sequences (*Bombyx mori* and *Spodoptera litura*) for which there is no current proof of enzymatic activity. The hydropathy plot (web.expasy.org/protscale/) indicates that CG9384 has a typical Golgi glycosyltransferase topology with a transmembrane domain (boxed in red) within the first 30 amino acids, immediately following a cytosolic dibasic motif. The N-glycosylation sites present solely in CG9384 or conserved in comparison to other sequences are boxed in green; the probable divalent cation binding EDD site is boxed in purple.

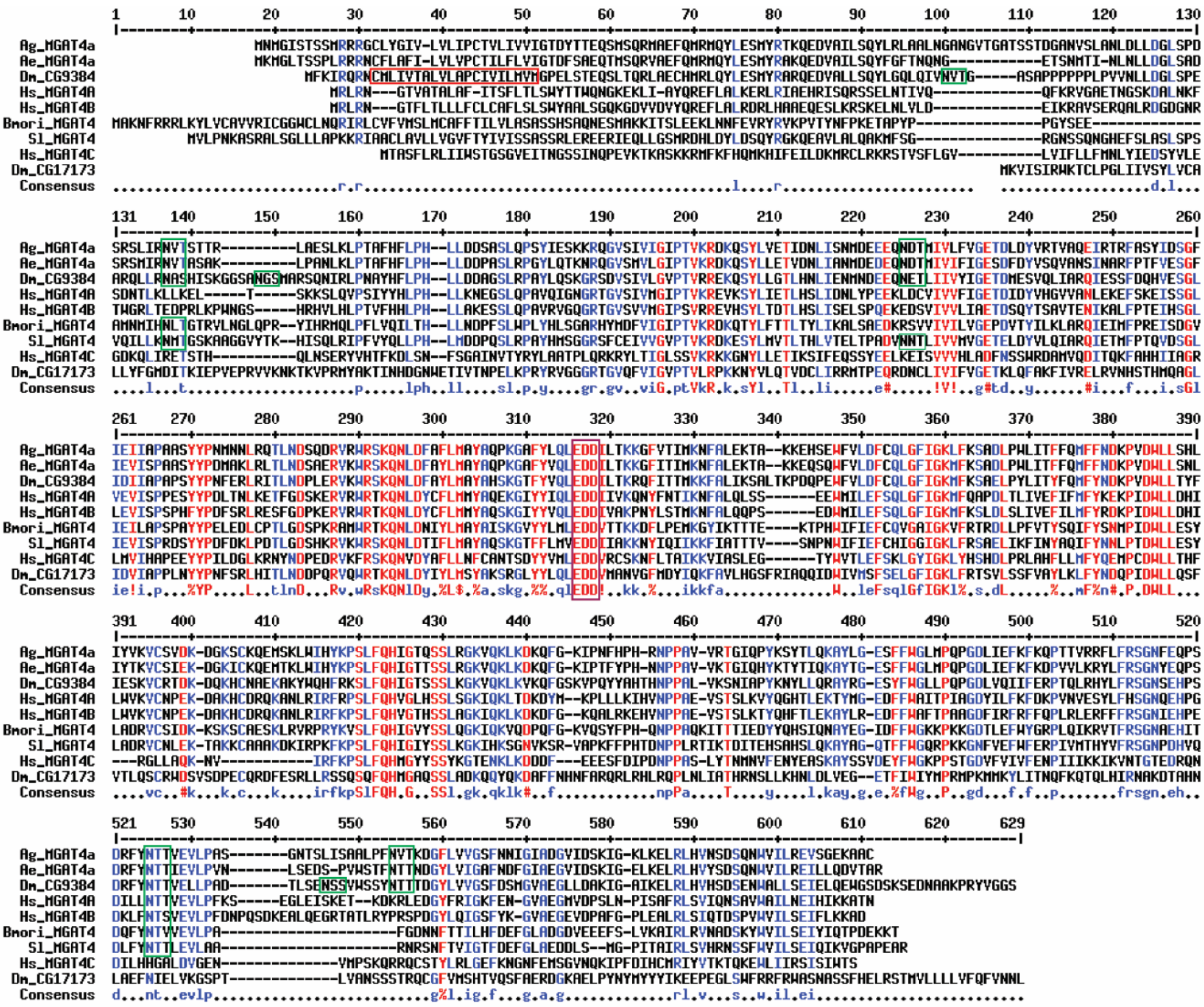

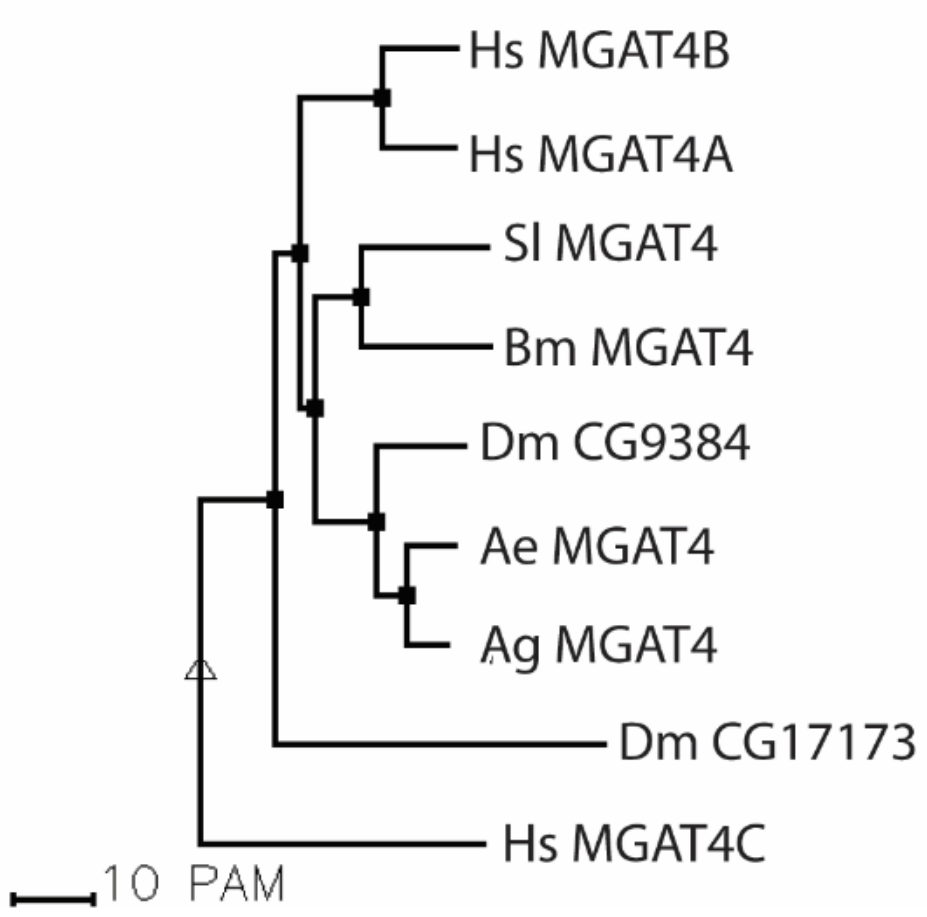

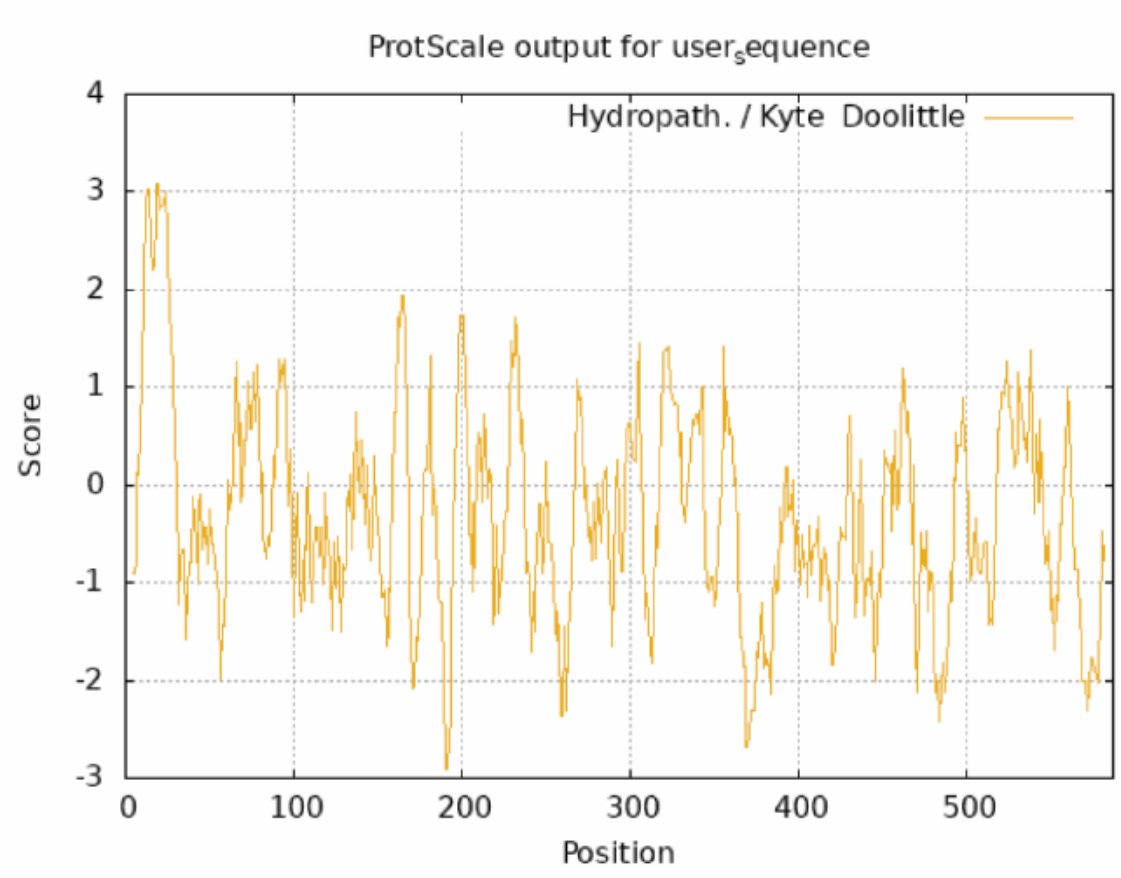

Supplementary Figure 8: Summary of structures of Drosophila N-glycans, O-glycans and glycosphingolipids and a potential N-glycan biosynthetic scheme. **(A)** Example structures for oligomannosidic, paucimannosidic, hybrid, pseudohybrid and triantennary complex N-glycans as observed in the current study are depicted. **(B)** Example structures of species-specific complex N-glycans from other insects as shown by our previous publications. **(C)** Examples of Drosophila O-glycans and glycosphingolipid structures as shown by previous publications. **(D)** Example steps for Drosophila N-glycan processing; gene names or abbreviated enzyme names are indicated, whereby those in red are hypothetical and the relevant genes/enzymes are unknown; FDL, fused lobes hexosaminidase CG8824; FucT6, core α1,6-fucosyltransferase CG2448; FucTA, core α1,3-fucosyltransferase CG6869; ManII, Golgi mannosidase II CG18802; MGAT1/2/4, *N*-acetylglucosaminyltransferases CG13431, CG7921 and CG9384; β3GalT, undefined GlcNAc-modifying β1,3-galactosyltransferase; β3GalT (CG33145), LacdiNAc-modifying β1,3-galactosyltransferase CG33145; β3GlcAT, β1,3-glucuronyltransferases CG3881 and CG6207; β4GalNAcT, GlcNAc-modifying β1,4-N-acetylgalactosaminyltransferase (CG8536); PET, undefined phosphoethanolaminyltransferases; SO_4T, undefined fucose and mannose-modifying sulphotransferases.

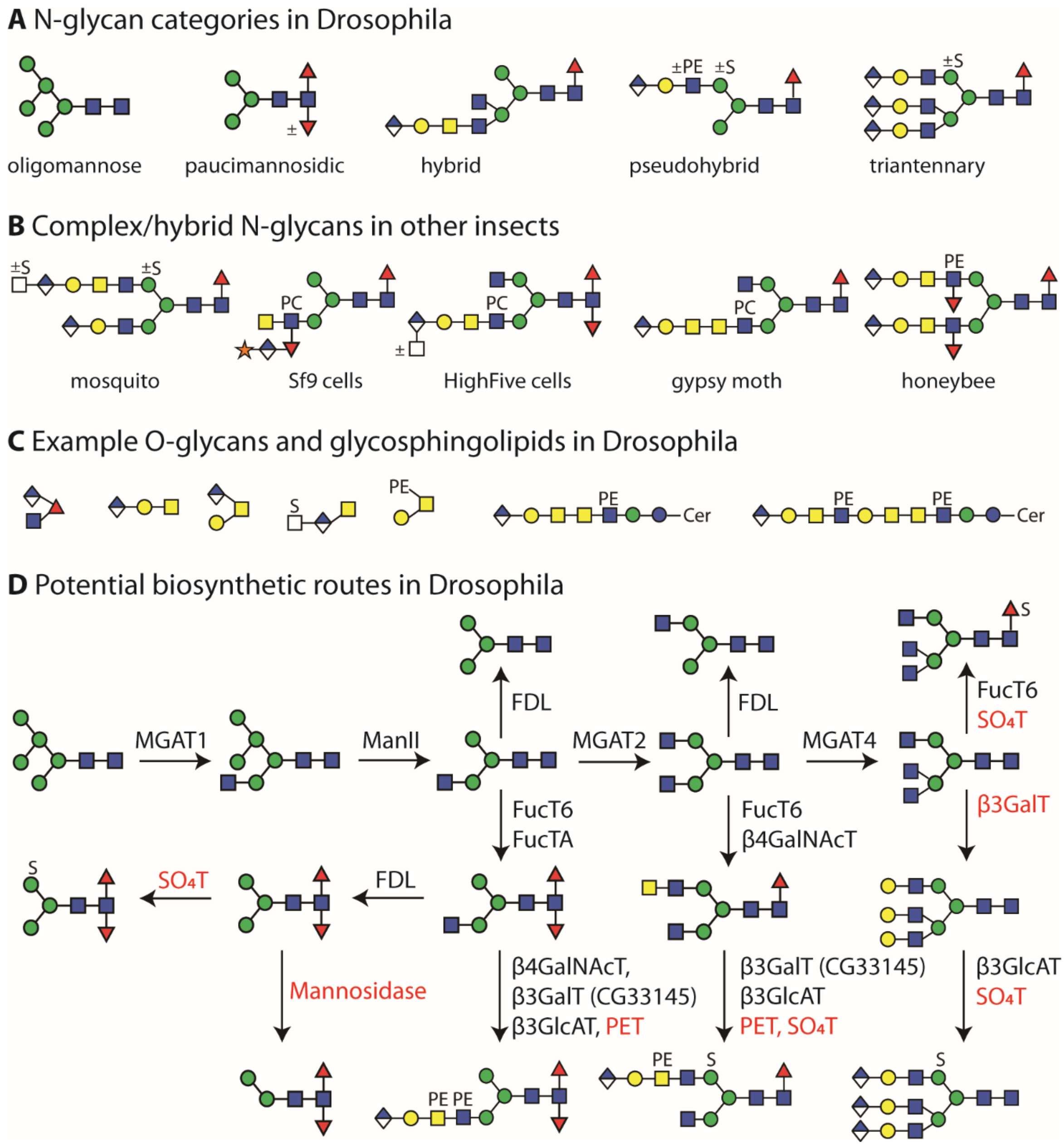

Supplementary Table: Proposed N-glycan structures for *Drosophila melanogaster* Tf24 adults. The glycans are ordered according to *m/z* for the pyridylaminated structure (as $[M+H]^+$ for most structures or $[M-H]^-$ for sulphated structures or $[M-2H+Na]^-$ for sulphated structures whose compositions are shown in red) with the neutral glycans followed by the anionic glycans. Given are abbreviated compositions (of the form $H_xN_yF_{0\text{-}2}U_{0\text{-}3}PE_{0\text{-}2}S_{0\text{-}2}$, i.e., $Hex_xHexNAc_yFuc_{0\text{-}2}GlcA_{0\text{-}3}PE_{0\text{-}2}S_{0\text{-}2}$), SNFG structures, RP-amide retention time in glucose units (g.u.), the GlyTouCan ID and the presence in the PNGase F/A-released body and/or head samples (see ***Figures 1 and 2*** in the main text) or in the head PNGase A-only sample (***Supplementary Figure 2***) or head PNGase F-only Kinetex (Kin) samples (***Figure 6*** in the main text).

m/z<br>Composition	Structure<br>Retention	Glucose units g.u.	GlyTouCanID	Presence<br>Body/Head/Kinetex (anionic)		
811.3<br>HN2F	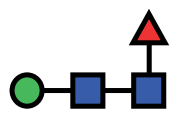	8.1-8.2	G32152BH	☒	☒	
827.3<br>H2N2	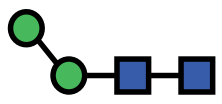	7.2-7.6	G22573RC	☒	☒	
957.3<br>H1N2F2	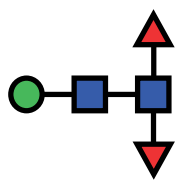	6.2-6.5	G92953ND	☒	☒	
973.3<br>H2N2F	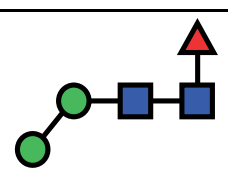	8.1-8.2	G42466VF	☒	☒	
	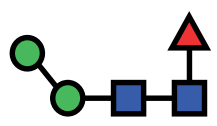	9.1-9.2	G00395TQ	☒	☒	
989.3<br>H3N2	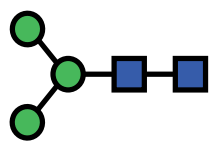	7.2-7.6	G22768VO	☒	☒	
1119.4<br>H2N2F2	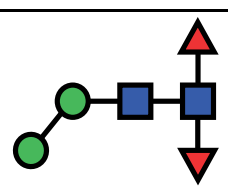	6.0	G17601US		A	
	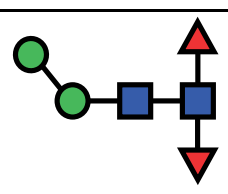	6.6-6.8	G88303DD	☒	☒	
1135.4<br>H3N2F	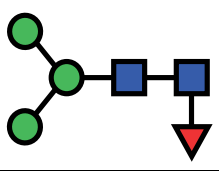	5.4-5.6	G43648DB	☒	☒	
	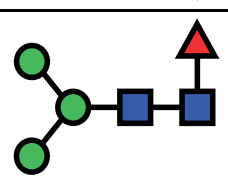	9.1-9.2	G82348BZ	☒	☒	
1151.4<br>H4N2	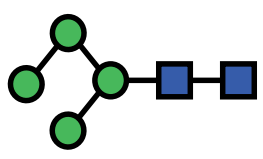	7.2-7.6	G09724ZC	☒	☒	
1176.4<br>H2N3F	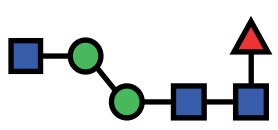	11.5	G39203UC	☒	☐	
1192.4<br>H3N3	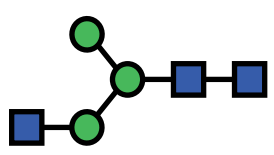	7.0	G61751GZ	☒	☒	
1281.5<br>H3N2F2	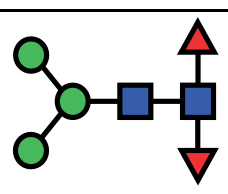	6.6-7.0	G11460AB	☒	☒	
1297.5<br>H4N2F	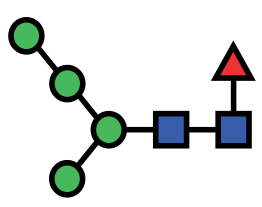	8.7-8.8	G27389SR	☒	☒	
	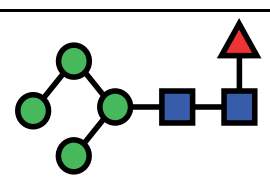	9.1-9.2	G32156ZV	☒	☒	

m/z Composition	Structure Retention	Glucose units g.u.	GlyTouCanID	Presence Body/Head/Kinetex (anionic)		
1313.4 H5N2	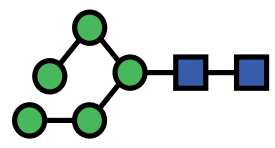	6.8	G07617FP	☒	☒	
	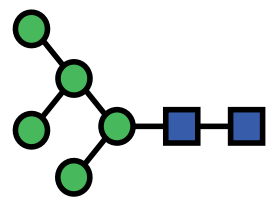	7.2-7.6	G55220VL	☒	☒	
1338.5 H3N3F	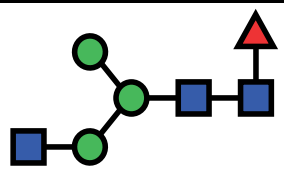	8.7-8.8	G07483YN	☒	☒	
	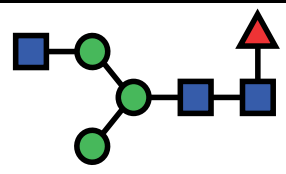	11.8	G45889JQ	☒	☒	
1354.5 H4N3	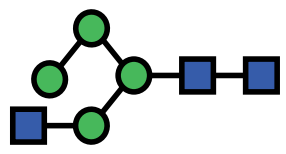	7.0-7.2	G53168IY	☒	☒	
1395.5 H3N4	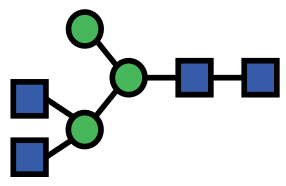	8.1-8.2	G92024SL	☒	☒	
	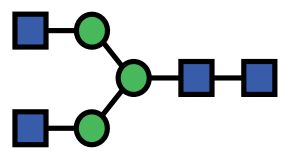	8.1-8.2	G39213VZ	☒	☒	
1459.5 H5N2F	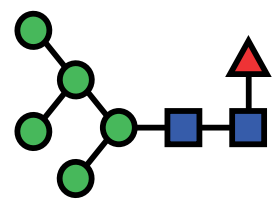	9.1-9.2	G99858XP	☐	☒	
1461.5 H3N3FPE	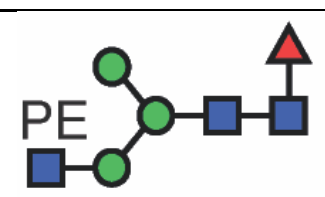	7.8-8.0	G05373KX	☒	☒	
1475.5 H6N2	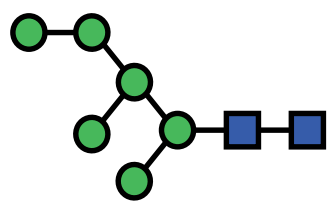	6.0-6.6	G61846BY	☒	☒	
	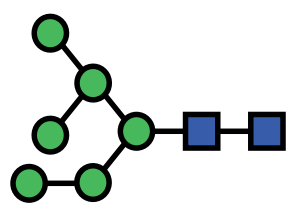	6.8	G80966KZ	☒	☒	
	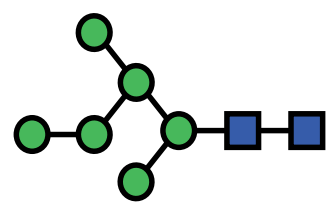	8.1-8.2	G34499SX	☒	☒	
1484.5 H3N3F2	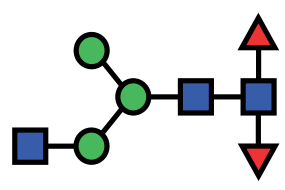	6.2-6.6	G41720HN	☒	☒	
	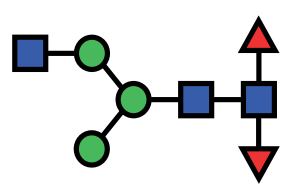	7.8-8.0	G89117DH	☒	☒	
1500.5 H4N3F	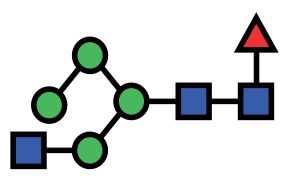	8.7-8.8	G58969RU	☒	☒	
	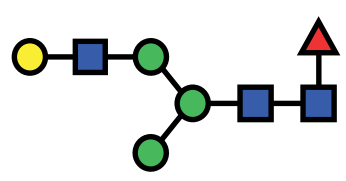	11.5	G99117SF	☒	☐	
1516.5 H5N3	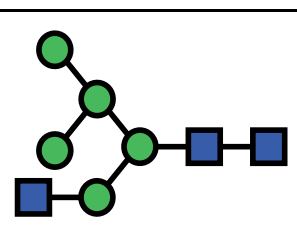	7.0-7.2	G08520NM	☒	☒	

m/z<br>Composition	Structure<br>Retention	Glucose<br>units g.u.	GlyTouCanID	Presence<br>Body/Head/Kinetex (anionic)		
1541.6<br>H3N4F	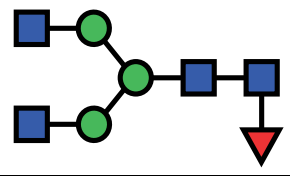	5.3	G64669AQ		A	
	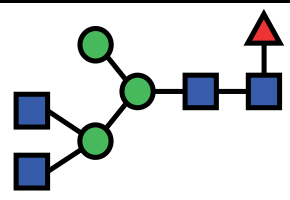	10.5	G26182BM	☒	☒	
	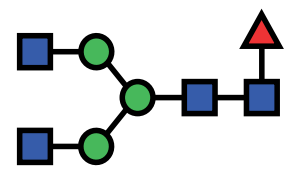	10.5	G80858MF	☒	☒	
1607.5<br>H3N3F2PE	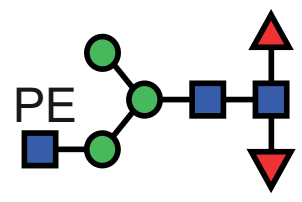	5.6	G50446OJ		A	
1637.6<br>H7N2	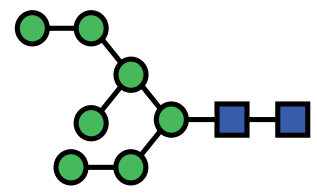	5.8-6.2	G83161QT	☒	☒	
	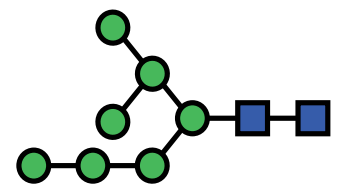	6.2-6.5	G68668TB	☒	☒	
	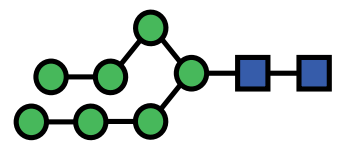	7.0-7.2	G04216GB	☒	☒	
	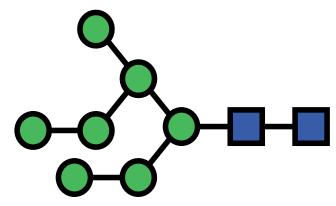	7.2-7.6	G63337SS	☒	☒	
1662.6<br>H5N3F	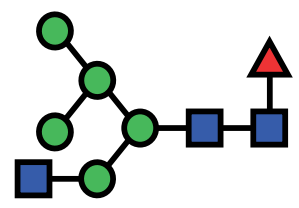	8.7-8.8	G82942ZJ	☒	☒	
	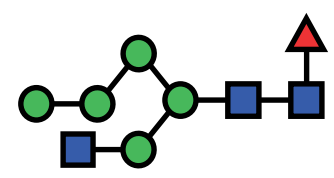	9.8-10.0	G82371GR	☒	☒	
1664.6<br>H3N4FPE	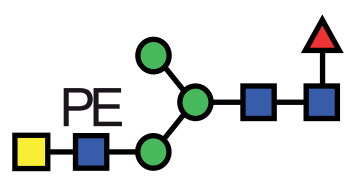	8.7-8.8	G48618AZ	☒	☒	
1687.6<br>H3N4F2	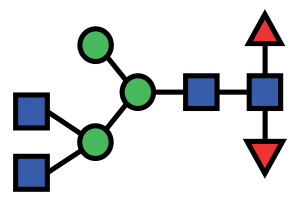	7.0	G38137TC		A	
1703.6<br>H4N4F	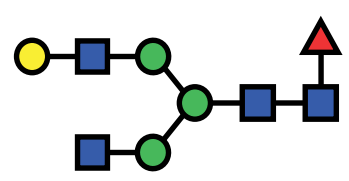	9.8-10.0	G08983AS	☒	☒	
1744.6<br>H3N5F	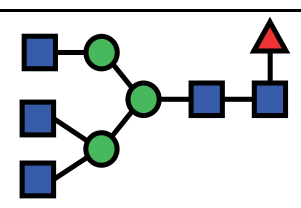	12.5	G61207RZ	☒	☒	
1799.6<br>H8N2	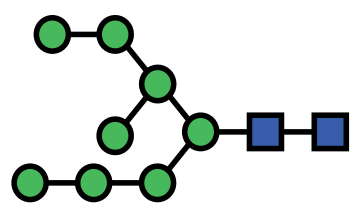	5.4-5.6	G40702WU	☒	☒	
	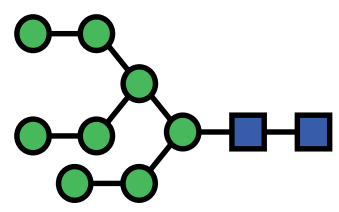	6.2-6.5	G89864BN	☒	☒	
	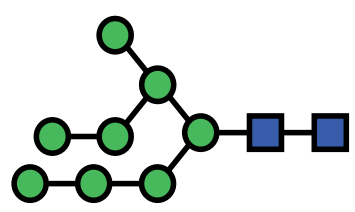	7.0	G91704UR	☒	☒	

m/z<br>Composition	Structure<br>Retention	Glucose<br>units g.u.	GlyTouCanID	Presence<br>Body/Head/Kinetex (anionic)		
1810.6<br>H3N4F2PE	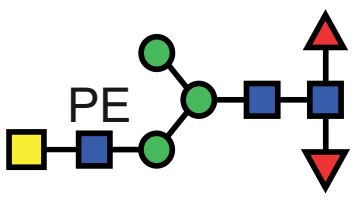	6.2	G15437OS		A	
1826.6<br>H4N4FPE	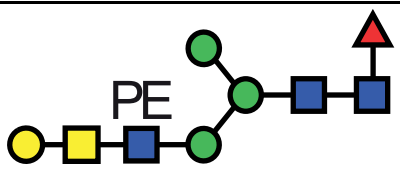	8.7-8.8	G75432VQ	☒	☒	
1961.7<br>H9N2	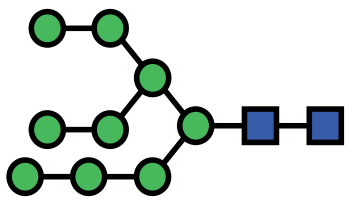	5.8-6.2	G60230HH	☒	☒	
	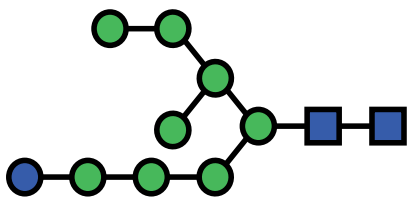	6.6	G81356NS	☐	☒	
	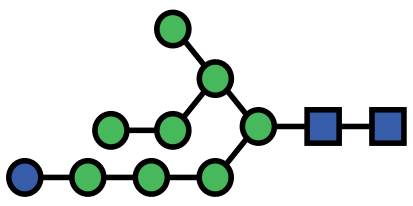	8.1-8.2	G39687WG	☒	☒	
1972.7<br>H4N4F2PE	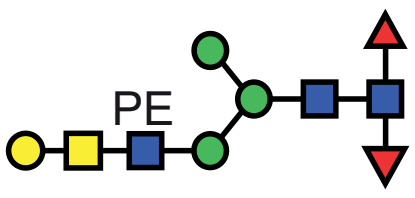	5.2	G29308XG		A	
2095.7<br>H4N4F2PE2	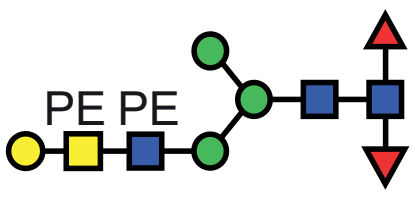	5.0	G25269BI		A	
2123.7<br>H10N2	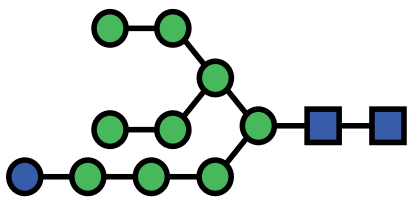	7.0-7.2	G19958IL	☒	☒	
889.3<br>HN2FS	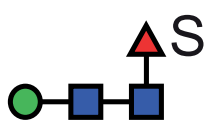	7.8-8.2	G31196LU	☒	☒	T
905.3<br>H2N2S	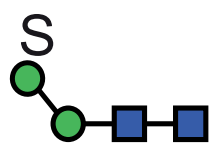	6.2-6.5	G55098YX	☒	☒	
1051.3<br>H2N2FS	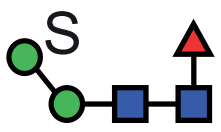	7.8-8.2	G44492SH	☒	☒	
	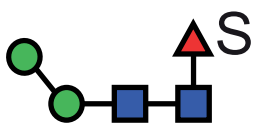	8.8-9.0	G31424LO	☒	☒	U
1067.3<br>H3N2S	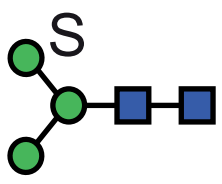	5.9-6.1	G52223DC	☒	☒	K
1213.4<br>H3N2FS	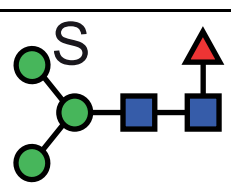	7.5-7.8	G30483QL	☒	☒	T
	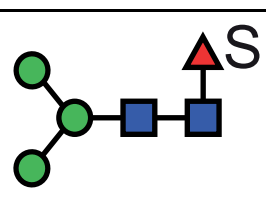	9.0-9.4	G34964EN	☒	☒	U
1254.4<br>H2N3FS	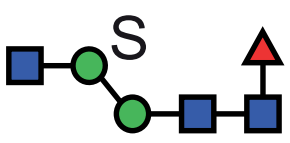	8.0-8.3	G95610WF	☒	☒	V
1270.4<br>H3N3S	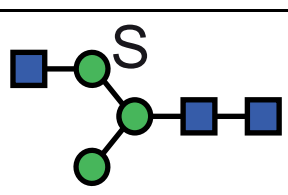	5.9-6.1	G77342IN	☒	☒	K

m/z<br>Composition	Structure<br>Retention	Glucose units g.u.	GlyTouCanID	Presence<br>Body/Head/Kinetex (anionic)		
1315.4<br>H3N2FS2 (Na)	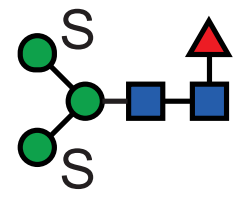		G18934GQ	☐	☐	N
1359.5<br>H3N2F2S	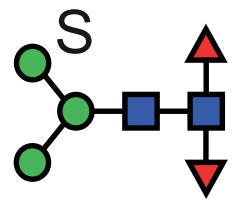	5.5-5.9	G01262MY	☒	☒	
1416.5<br>H3N3FS	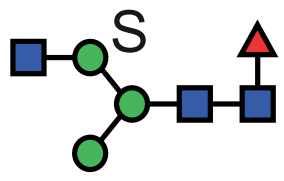	7.5-7.8	G19484FC	☒	☒	T
	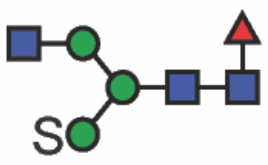		G50000VP	☐	☐	U
	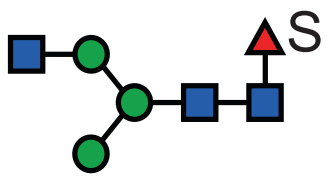	11.4-12.0	G51521AG	☒	☒	X
1473.5<br>H3N4S	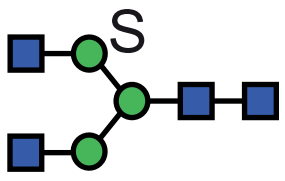	5.9-6.1	G04709HD	☒	☒	L
1518.5<br>H3N3FS2 (Na)	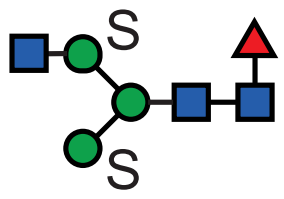		G29659RI	☐	☐	N
1530.5<br>H4N3U	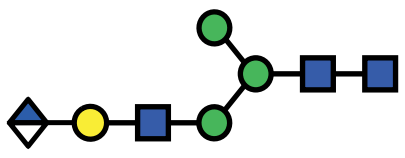	4.6-4.8	G54692DX	☒	☒	G
	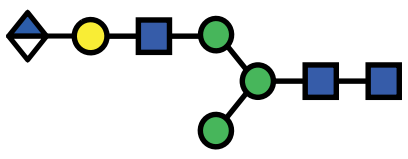	7.2-7.4	G86956LZ	☒	☐	O
1562.5<br>H3N3F2S	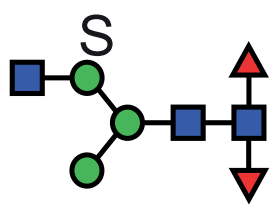	5.8-6.1	G31436MH	☒	☒	
1578.5<br>H4N3FS	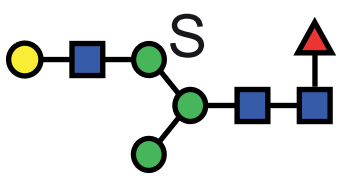	8.5-8.8	G28222ZS	☒	☒	U
1608.6<br>H4N3US	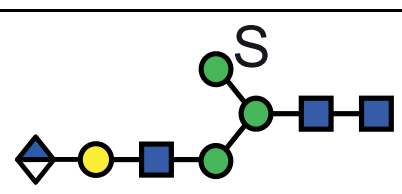	4.1-4.3	G99345CZ	☒	☒	E
	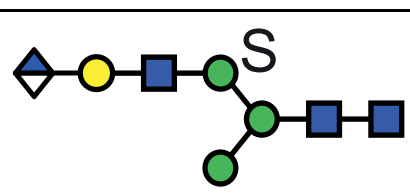	5.4-5.6	G38579FE	☒	☒	H
1619.6<br>H3N4FS	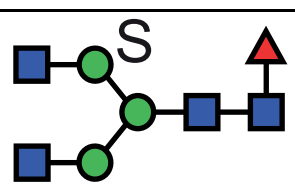	7.6-7.8	G65121QQ	☒	☒	U
	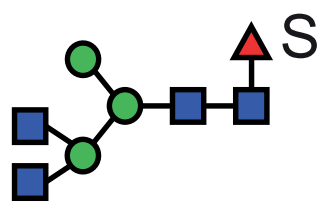	10.0-10.5	G81067EB	☒	☒	V
1653.5<br>H4N3UPE	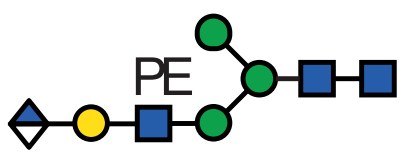		G33620OQ	☐	☐	D
1676.6<br>H4N3FU	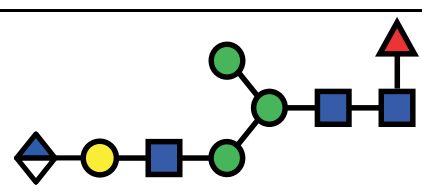	6.3-6.7	G29596AF	☒	☒	P
	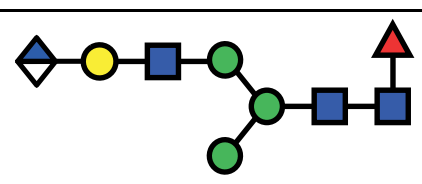	8.8-9.0	G91205AM	☒	☒	V

m/z Composition	Structure Retention	Glucose units g.u.	GlyTouCanID	Presence Body/Head/Kinetex (anionic)		
1676.6 H3N5S	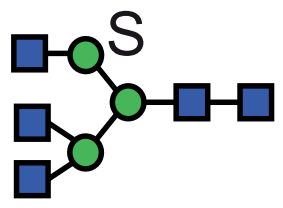	7.8	G12052NI	☒	☒	Q
1692.6 H5N3U	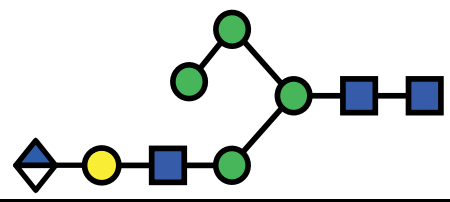	4.6-4.8	G56489TJ	☒	☐	G
1731.5 H4N3UPES	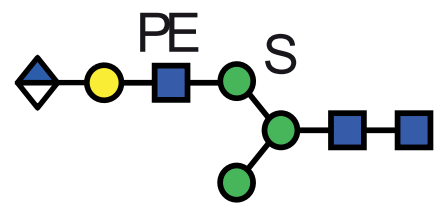	4.0-4.5	G72235YA	☒	☐	E
1733.6 H4N4U	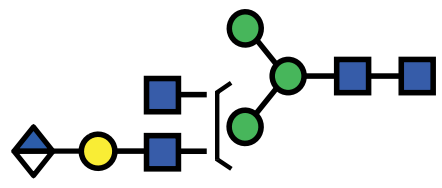	5.4-5.8	G41334WT	☒	☒	H
	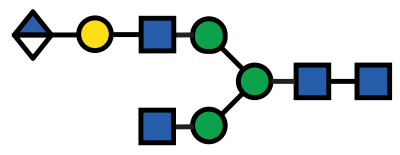		G16033BA	☐	☐	M
	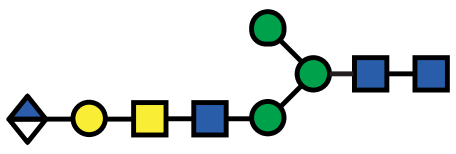	6.3-6.5	G76402LV	☒	☒	O
1754.6 H4N3FUS	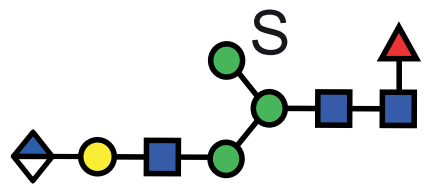	5.6	G51915FS	☒	☒	J
	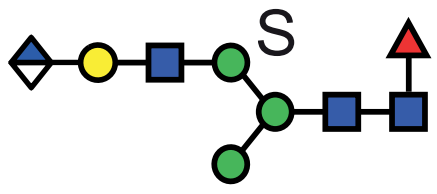	6.9-7.1	G98014ZH	☒	☒	Q
1765.6 H3N4F2S	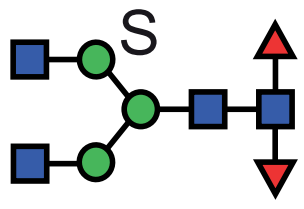	5.8-6.1	G89055YF	☒	☒	
1781.6 H4N4FS	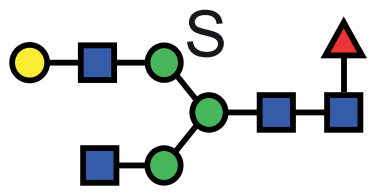	8.5-8.8	G16314UZ	☒	☒	
	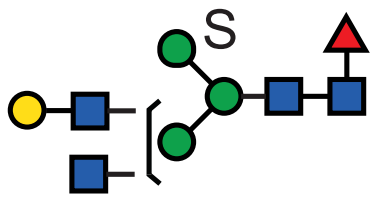		G49106IM	☐	☐	T
1799.6 H4N3FUPE	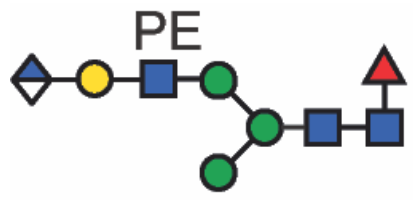		G09040SF	☐	☐	R
1811.6 H4N4US	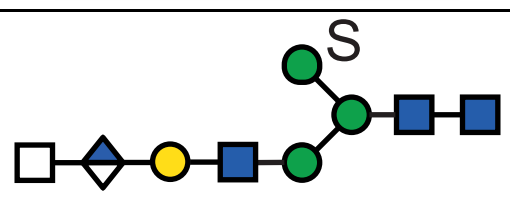		G78563VK	☐	☐	D
	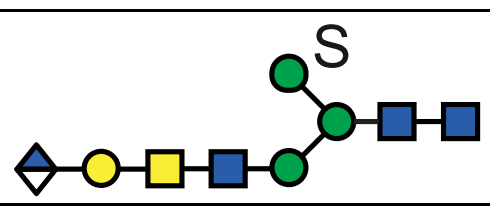	4.0-4.5	G43789PN	☒	☐	
	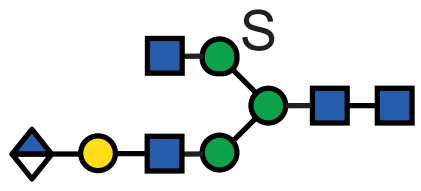		G22947IA	☐	☐	E
	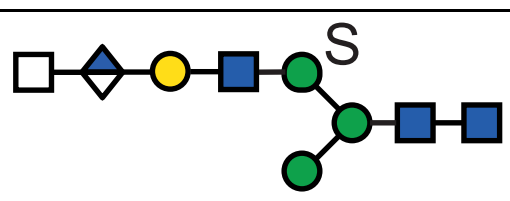		G37135XE	☐	☐	G
	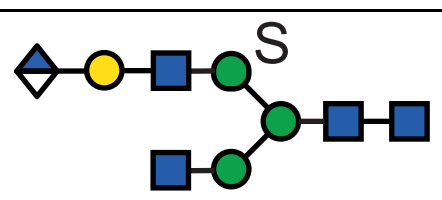		G22238MS	☐	☐	J

m/z<br>Composition	Structure<br>Retention	Glucose units g.u.	GlyTouCanID	Presence<br>Body/Head/Kinetex (anionic)		
1822.6<br>H4N3F2U	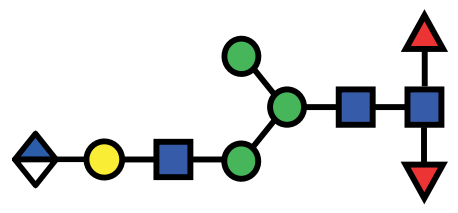	4.6-5.0	G98578BX	☐	☒	
1822.6<br>H3N5FS	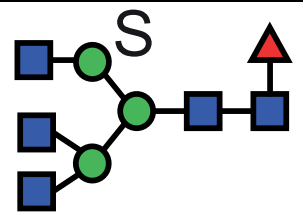	10-10.5	G04850XN	☒	☒	W
	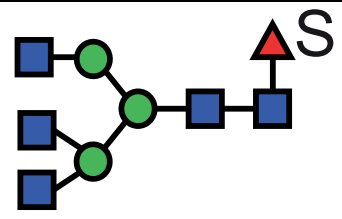	12.0-12.5	G39857GV	☐	☒	
1854.6<br>H6N3U	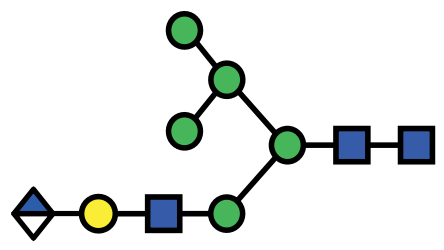	4.6-4.8	G36790MW	☒	☐	
1856.6<br>H4N4UPE	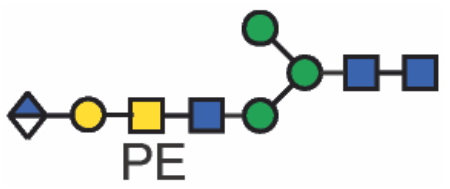	4.0-4.5	G54837DP	☒	☐	J
1877.6<br>H4N3FUPES	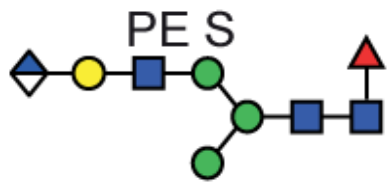	4.9-5.1	G27895BU	☒	☒	K
1879.6<br>H4N4FU	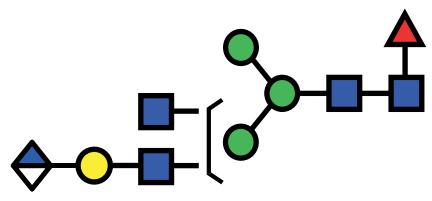	6.9-7.1	G84232UE	☒	☒	Q
	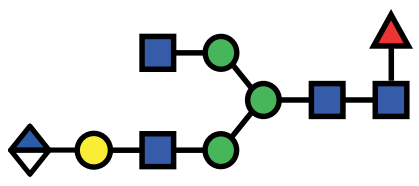	7.5-7.8	G77138KY	☒	☒	S
	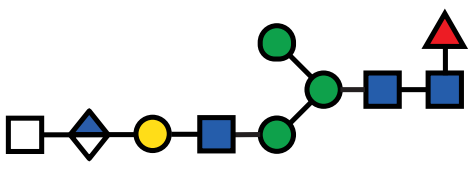		G08644BJ	☐	☐	T
	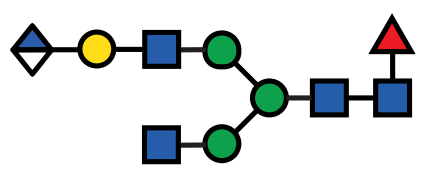		G19829IP	☐	☐	U
	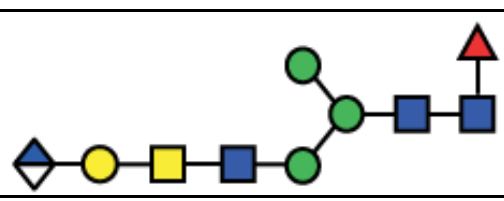	8.0-8.3	G19590UM	☒	☒	V
	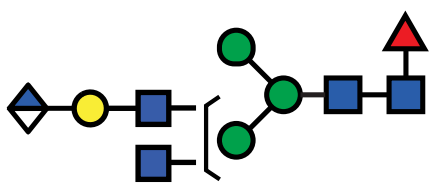	8.8-9.2	G85447QK	☒	☒	
	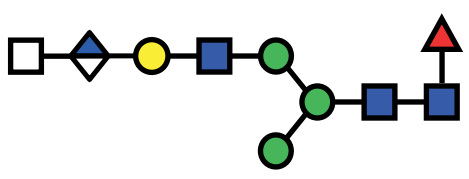	10	G88402LF	☐	☒	
	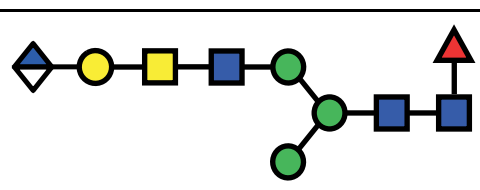	12.0-12.5	G70363TC	☐	☒	
1895.6<br>H5N4U	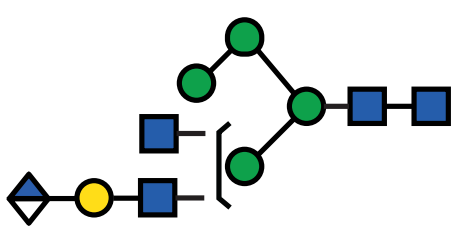		G37093NN	☐	☐	G
1924.6<br>H3N5FS2 (Na)	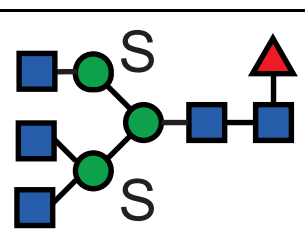		G24333SF	☐	☐	M

m/z Composition	Structure Retention	Glucose units g.u.	GlyTouCanID	Presence Body/Head/Kinetex (anionic)		
1934.6 H4N4UPES	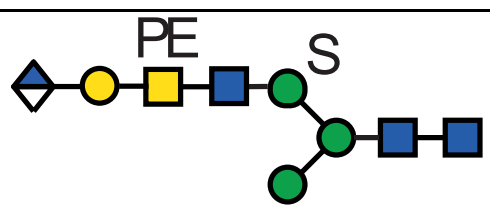		G34152VR	☐	☐	E
1936.7 H4N5U	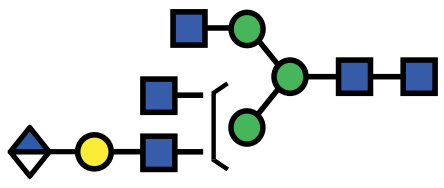	6.3-6.7	G75081GZ	☒	☒	K
1943.7 H5N4FS	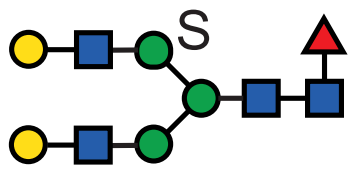		G59011ZC	☐	☐	T
1957.6 H4N4FUS	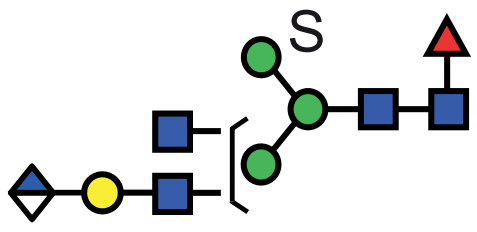	5.0-5.5	G32764WT	☒	☒	J
1957.6 H4N4FUS	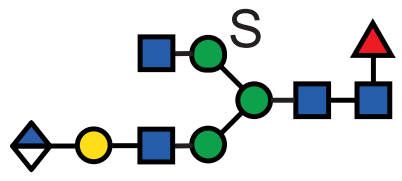		G27811QZ	☐	☐	K
	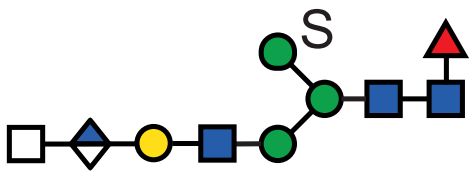		G58571HV	☐	☐	N
	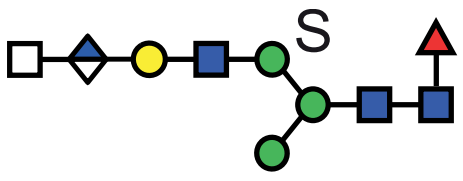	7.2-7.4	G64550UU	☒	☐	O
	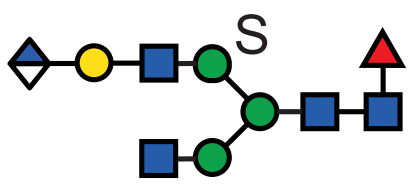		G58919WJ	☐	☐	R
1979.6 H4N4UPE2	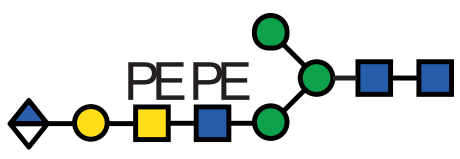		G71646BR	☐	☐	J
2002.7 H4N4FUPE	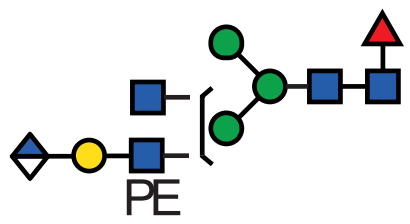		G39660AL	☐	☐	L
	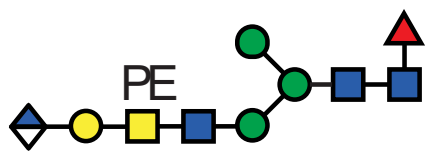	6.3-6.7	G78164WO	☒	☒	T
	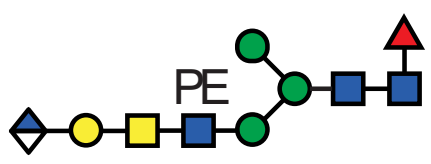	7.5-7.8	G95814IX	☒	☒	V
2014.7 H4N5US	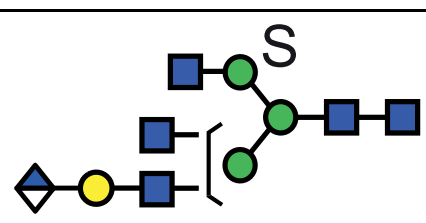	4.5-4.8	G37880VK	☒	☒	F
2025.7 H4N4F2U	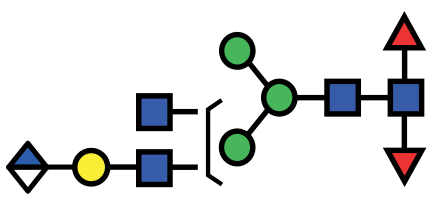	5.4-5.8	G91265VI	☒	☒	
	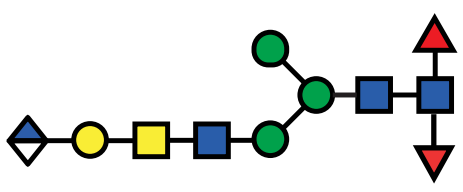	5.8-6.0	G92006ZJ	☐	☒	

m/z Composition	Structure Retention	Glucose units g.u.	GlyTouCanID	Presence Body/Head/Kinetex (anionic)		
2041.7 H5N4FU	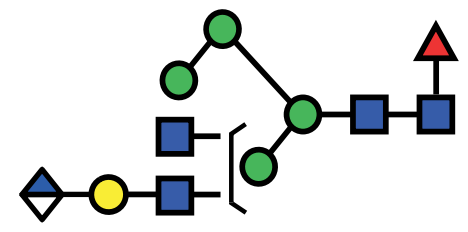	6.9-7.1	G95234IU	☒	☐	P
2059.7 H4N5UPE	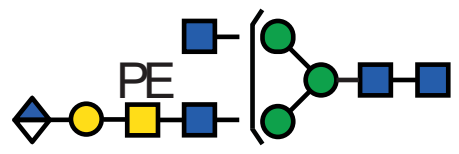		G29699UA	☐	☐	G
2071.7 H5N4U2	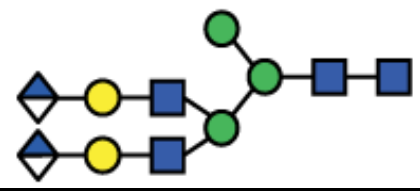	4.5-4.8	G92127XG	☒	☒	F
2080.7 H4N4FUPES	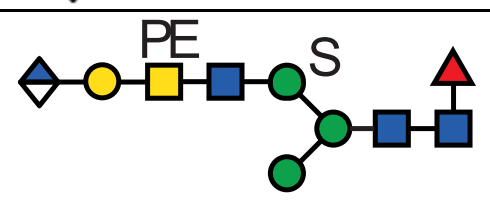		G13582ET	☐	☐	J
	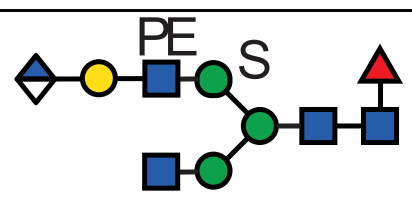		G17899EY	☐	☐	M
2082.7 H4N5FU	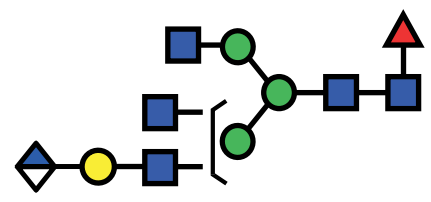	8.0-8.3	G68310RE	☒	☒	T
	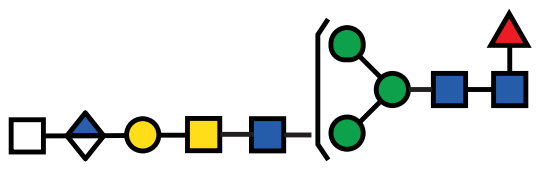		G87814CK	☐	☐	U
	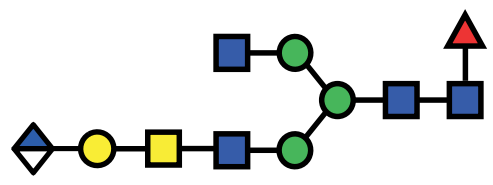	9.0-9.4	G66665WQ	☒	☒	X
	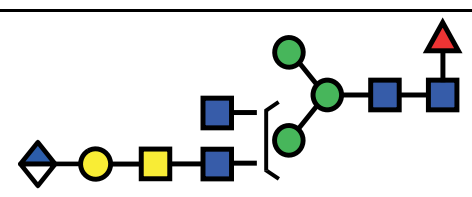	10-10.5	G49203RR	☒	☒	
2125.7 H4N4FUPE2	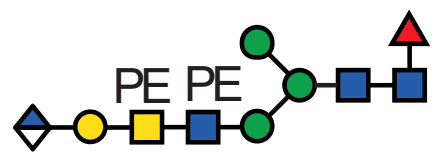		G82796OT	☐	☐	S
2146.7 H5N5FS	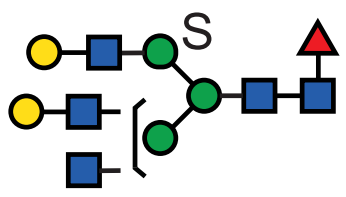		G92344LU	☐	☐	U
2148.7 H4N4F2UPE	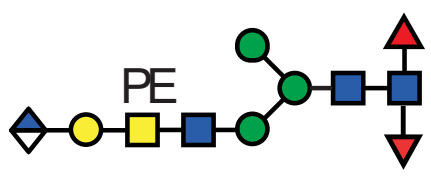	4.5-4.8	G47642FG	☐	☒	
2149.7 H5N4U2S	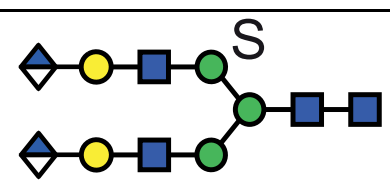	4.0-4.4	G23703HE	☐	☒	D
2160.7 H4N5FUS	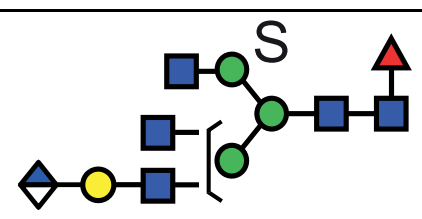	5.9-6.5	G16423YR	☒	☒	L
	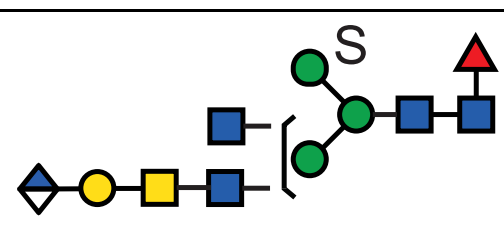		G95199WE	☐	☐	O
2194.7 H5N4U2PE	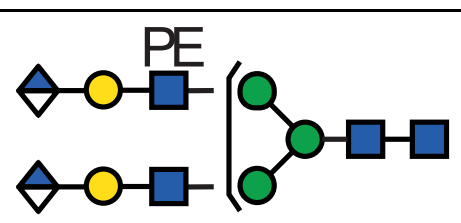		G13420ZM	☐	☐	E
2203.8 H6N4FU	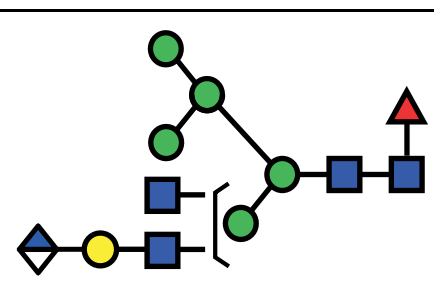	6.9-7.1	G27749GB	☒	☐	

m/z<br>Composition	Structure<br>Retention	Glucose<br>units g.u.	GlyTouCanID	Presence<br>Body/Head/Kinetex (anionic)		
2205.7<br>H4N5FUPE	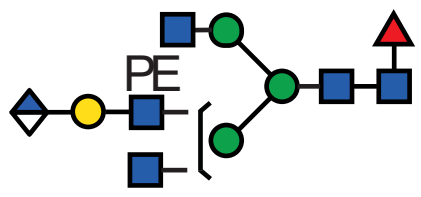		G23535HQ	☐	☐	O
	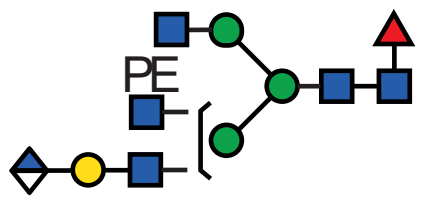		G15553DS	☐	☐	Q
	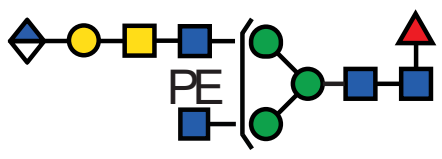		G21843LL	☐	☐	T
	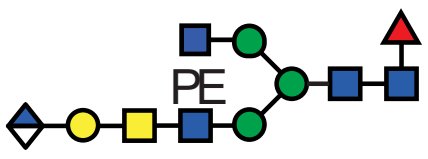	8.8-9.0	G22516RX	☒	☒	X
2217.7<br>H5N4FU2	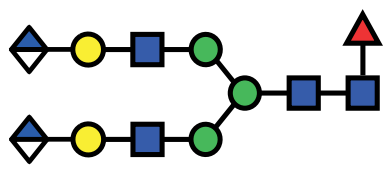	6.0	G47159JX	☐	☐	Fly
	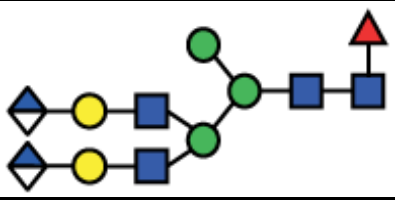	6.0-6.5	G53301DO	☒	☒	L
2271.7<br>H4N4F2UPE2	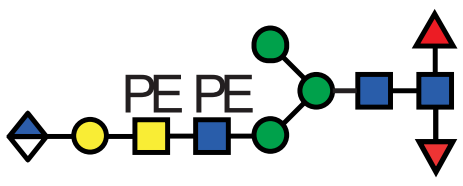	4.4-4.6	G68939OH	☐	☒	
2272.7<br>H5N4U2PES	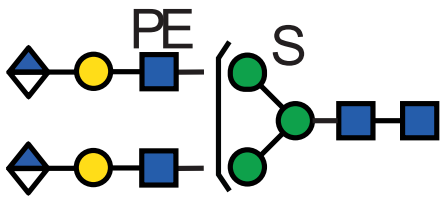		G25927EP	☐	☐	B
2274.8<br>H5N5U2	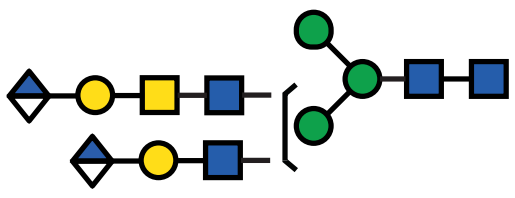		G15046AZ	☐	☐	G
2283.7<br>H4N5FUPES	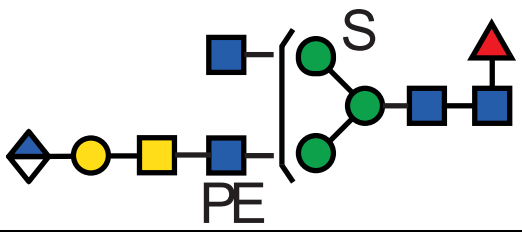		G95026UW	☐	☐	P
2285.9<br>H4N6FU	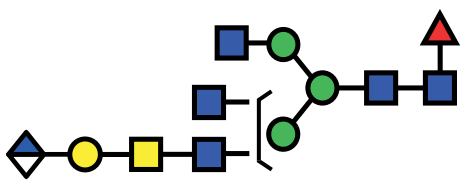	11.0-12.0	G16587XU	☐	☒	Y
2295.7<br>H5N4FU2S	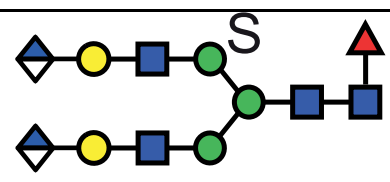	4.5-5.0	G47591AR	☒	☒	H
2317.7<br>H5N4PE2U2	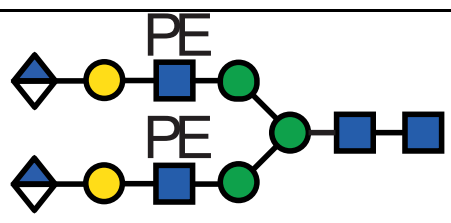		G85357CX	☐	☐	B
2328.7<br>H4N5UFPE2	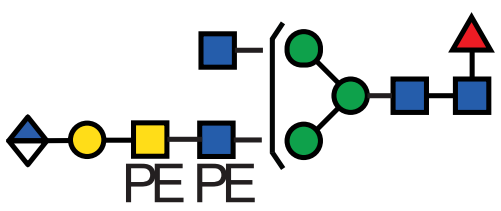		G97940BQ	☐	☐	U
2352.8<br>H5N5U2S	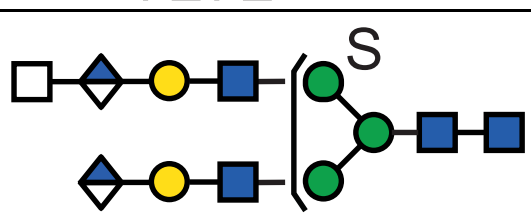		G94701XZ	☐	☐	C
	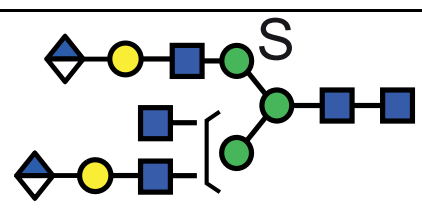	4.0-4.4	G57085PK	☒	☒	E

m/z Composition	Structure Retention	Glucose units g.u.	GlyTouCanID	Presence Body/Head/Kinetex (anionic)		
2363.8 H4N6FUS	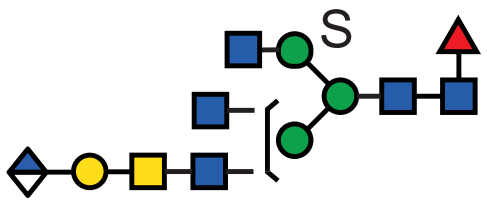		G35773DM	☐	☐	R
2395.7 H5N4U2PE2S	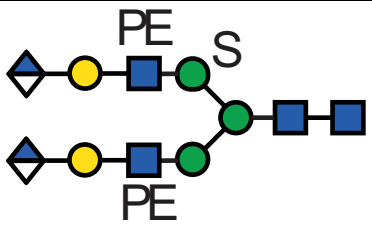		G03777ZS	☐	☐	A
2397.8 H5N5U2PE	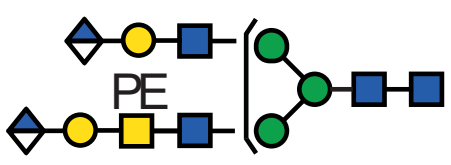		G27488MM	☐	☐	B
	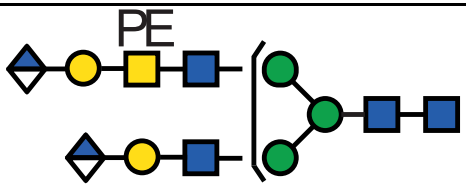		G91210JW	☐	☐	F
2420.8 H5N5FU2	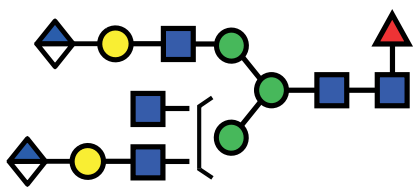	6.4-6.6	G94787IB	☒	☒	M
	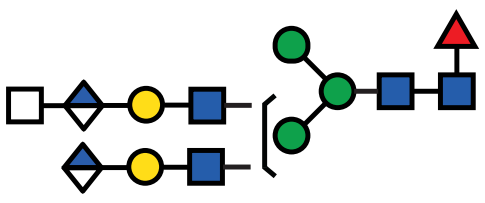		G36617GL	☐	☐	N
	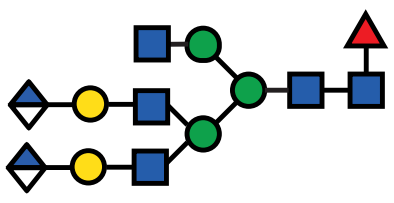		G53929PJ	☐	☐	P
2463.7 H5N4FU2PE2	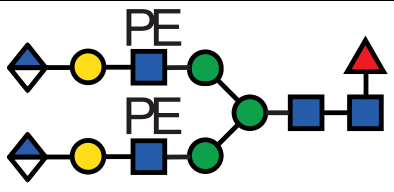		G78524YE	☐	☐	C
2498.8 H5N5FU2S	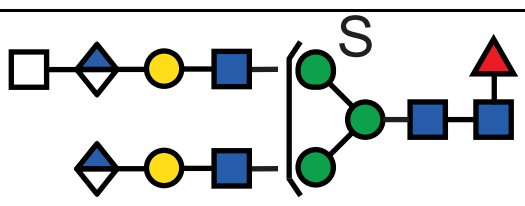		G95275OR	☐	☐	G
	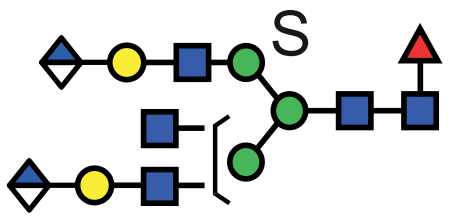	5.5-6.0	G55528OI	☒	☒	J
2520.8 H5N5U2PE2	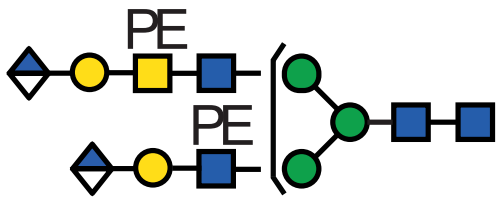		G40305TK			C
2598.8 H5N5U2PE2S	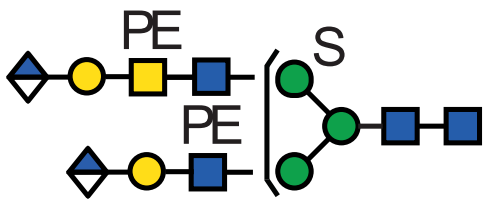		G55724RV	☐	☐	A
2690.8 H6N5U3S	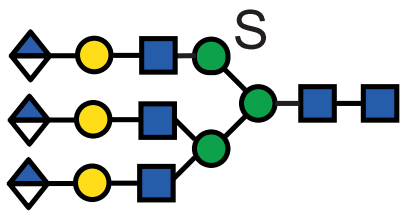		G52866YM	☐	☐	C
2701.9 H5N6FU2S	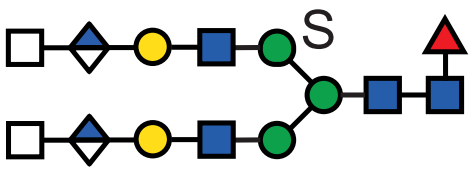		G14681FK	☐	☐	H
2758.9 H6N5FU3	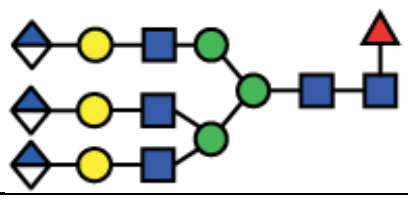	6.2	G80295RC	☐	☒	J
2836.9 H6N5FU3S	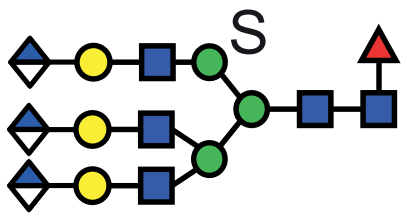	4.5-4.8	G17510TB	☐	☒	G

m/z<br>Composition	Structure<br>Retention	Glucose<br>units g.u.	GlyTouCanID	Presence<br>Body/Head/Kinetex (anionic)		
2893.9<br>H6N6U3S	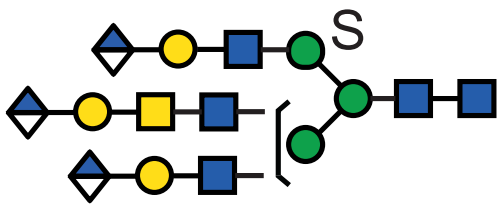		G72619WJ	☐	☐	D
3040.0<br>H6N6FU3S	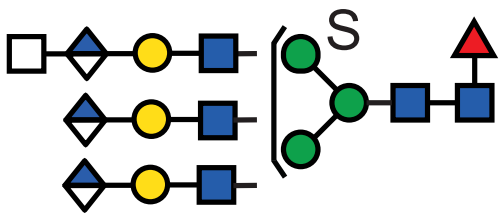		G10871WD	☐	☐	F
